# *Schizophyllum commune* has an extensive and functional alternative splicing repertoire

**DOI:** 10.1038/srep33640

**Published:** 2016-09-23

**Authors:** Thies Gehrmann, Jordi F. Pelkmans, Luis G. Lugones, Han A. B. Wösten, Thomas Abeel, Marcel J. T. Reinders

**Affiliations:** 1 Delft Bioinformatics Lab, Delft University of Technology, Delft, Zuid-Holland 2628 CD, The Netherlands; 2 Microbiology, Department of Biology, Utrecht University, Utrecht, Utrecht 3585 CH, The Netherlands; 3 Broad Institute of MIT and Harvard, Cambridge, Massachusetts MA02142, United States of America

## Abstract

Recent genome-wide studies have demonstrated that fungi possess the machinery to alternatively splice pre-mRNA. However, there has not been a systematic categorization of the functional impact of alternative splicing in a fungus. We investigate alternative splicing and its functional consequences in the model mushroom forming fungus *Schizophyllum commune*. Alternative splicing was demonstrated for 2,285 out of 12,988 expressed genes, resulting in 20% additional transcripts. Intron retentions were the most common alternative splicing events, accounting for 33% of all splicing events, and 43% of the events in coding regions. On the other hand, exon skipping events were rare in coding regions (1%) but enriched in UTRs where they accounted for 57% of the events. Specific functional groups, including transcription factors, contained alternatively spliced genes. Alternatively spliced transcripts were regulated differently throughout development in 19% of the 2,285 alternatively spliced genes. Notably, 69% of alternatively spliced genes have predicted alternative functionality by loss or gain of functional domains, or by acquiring alternative subcellular locations. *S. commune* exhibits more alternative splicing than any other studied fungus. Taken together, alternative splicing increases the complexity of the *S. commune* proteome considerably and provides it with a rich repertoire of alternative functionality that is exploited dynamically.

Alternative splicing is an important regulatory mechanism, which in many eukaryotes provides additional complexity to the proteome required to sustain cell functions^1^. In humans, the overwhelming majority of genes are alternatively spliced, and mutations in alternative transcripts can give rise to diseases such as cancer^2^. Alternative splicing events have also been shown to contribute to the evolution of new species^3^.

Splicing results in removal of introns in pre-mRNAs, and alternative splicing gives rise to different mRNA variants of a gene. All resulting mRNAs consist of a coding region flanked by UnTranslated Regions (UTRs). We distinguish primitive events, i.e. basic splice operations which only affect a single transcript, from composite events that relate to observed primitive events over multiple transcripts from the same gene (Fig. 1a,b). These events represent regions of especially high sequence heterogeneity between transcripts. Alternative splicing events occur not only in coding regions but also in UTRs, resulting in transcripts with alternative coding regions. Consequently, alternative splicing increases the complexity of the proteome which may also result in functional changes of the proteome when, for example, alternative splicing results in gain or loss of protein domains and functional elements. Alternative splicing events may be triggered by changes in environmental conditions^4^ or are part of developmental pathways^5^. On the other hand, alternative splicing may also be an error of the splicing machinery and not necessarily lead to alternative functionality. For instance, *RPL30* of *Saccharomyces cerevisiae* produces a transcript that fails to undergo splicing and later decays^6^.

Alternative splicing has been shown to occur in fungi. The first reported example of alternative splicing was in the *glaA* gene of *Aspergillus niger*, resulting in two isoforms of the glucoamylase enzyme^7^. Since then, other alternative splicing events have been described. For instance, the primary transcript of *ZAS1*+ of *Schizosaccharomyces pombe* contains two zinc finger domains, while its secondary transcript contains three of these domains^8^. Genome-wide comparative studies have shown that alternative splicing is common in most fungi, but that many yeasts exhibit few alternative splicing events^9^,^10^. Developmentally more complex fungi appear to have more alternative splicing (see Fig. 1e, where we compared the phylogeny of fungal species with reported richness of their alternative splicing). Genome-wide studies also showed that development impacts alternative splicing in *Fusarium graminearum*^11^, while growth medium composition impacts splicing in *Trichoderma longibrachiatum*^12^. In all studies, intron retentions were the most common primitive splicing events.

Alternative splicing at a genome-wide scale can be detected from expressed sequence tags^9^,^10^. With the advent of Next Generation Sequencing, detecting alternative splicing in fungi is mostly done by splice junction analysis. By comparing splice junctions that appear as gaps in the alignment of short reads to a reference genome, primary splicing events can be detected^12^. To reconstruct the structure of alternative transcripts, probabilistic modeling methods are used, which are based on primary event co-occurrence in single reads^13^,^14^. Along with splice junction analysis, this approach has also been separately used to study alternative splicing in fungi^11^. However, the high gene density and polyscystronic transcripts in many fungal genomes^15^, make this approach inapplicable. Gene UTRs overlapping with transcripts of neighboring genes cause the transcript reconstruction methods to erroneously fuse transcripts from different genes together (for more details see Supplementary Note S2). This shortcoming has prevented a systematic characterization of the alternative splicing repertoire for fungi with gene-dense genomes.

Despite the existing work to identify alternative splicing in fungi, a systematic analysis of the functional impact of alternative splicing is missing. In this work, we show the potential functional impact of alternative splicing in the mushroom forming fungus *Schizophyllum commune*. We designed a region-restricted probabilistic modeling method to detect alternative splicing for the gene-dense genome of *S. commune*. We detected alternative splice variants from RNA-seq data at different developmental stages of *S. commune*. We further analyzed the functional impact of alternative splicing and show its relationships with transcriptional regulation, subcellular localization, and alternative use of functional domains.

## Results

### Alternative splicing is a common mechanism in *S. commune*

**Figure 1:**
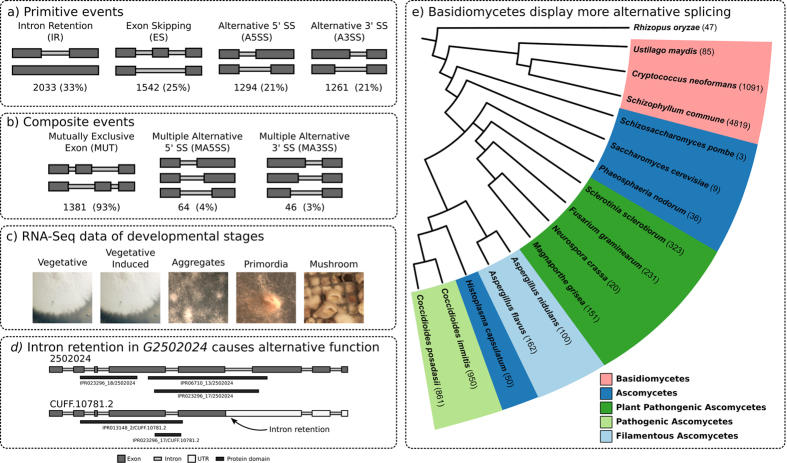
Alternative splicing during development of *S. commune*. (**a**) Primitive alternative splicing events that affect a single transcript. Two alternative transcripts are shown above each other. Thick dark gray bars represent exons, and skinny light gray bars indicate introns. The frequencies of each event in S. commune are provided beneath the figure. An intron retention (IR) occurs when an intron is not spliced out, while an exon skipping (ES) event splices out an exon. An alternative 5′ splicing site (A5SS) or a 3′ splicing site (A3SS) is characterized by an alternative splicing site on the 5′ end or the 3′ end of an exon, respectively. (**b**) Composite splicing events emerge from splicing events across multiple alternative transcripts. A mutually exclusive exon is when two (or more) transcripts skip different exons such that the two exons never occur together in a single transcript. Multiple alternative 5- and 3′ splicing sites (MA5SS and MA3SS, respectively) are when alternative transcripts have more than two alternative 5′ or 3′ splicing sites. (**c**) Alternative splicing was analysed using RNA-seq data from five developmental stages of the mushroom-forming *Schizophyllum commune* fungus. (**d**) An example of alternative splicing in gene *G2502024*. An intron retention (indicated by an arrow) introduces a premature stop codon. Due to this, the alternative transcript CUFF.10781.2 loses two protein domain annotations. (**e**) *S. commune* exhibits more alternative splicing than any previously studied fungus. Basidiomycetes are indicated in red, while different groups ascomycetes are indicated in blue and green. More phenotypically complex fungi have more alternative splicing (See Supplemental Note S1).

Sexual development of *S. commune* proceeds via different stages. Dikaryotic colonies resulting from mating of two compatible partners grow symmetrical in the dark and at high CO2 conditions. Asymmetrical growth and the formation of aggregates is induced when the colony is exposed to light and ambient CO2. The aggregates further develop into primordia and mature mushrooms. mRNA of five different stages of development of *S. commune* (Fig. 1c) was sequenced to study the incidence and functional impact of alternative splicing. In addition, RNA was used from deletion strains that are affected at different stages of mushroom formation^16^,^17^,^18^ (see Materials and Methods).

**Figure 2:**
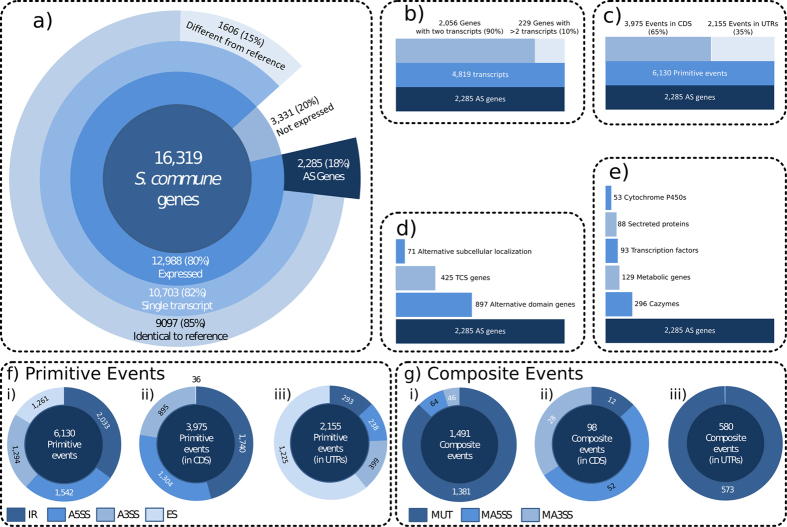
Statistics of alternative splicing. (**a**) The extend of alternative splicing revealed by analysing the RNA-seq data over different developmental stages of *Schizophyllum commune*. (**b**) The distribution of transcripts per gene. (**c**) The number of alternative splicing events within and outside of coding regions. (**d**) The number of genes that are functionally impacted by the alternative splicing on domains, subcellular localization and development. (**e**) The amount of alternative spliced genes for a number of functional groups. (**f**) The proportion of primitive events (i) genome wide, (ii) within coding regions, and (iii) in UTRs (Supplemental Note S10). (**g**) The proportion of composite events (i) genome wide, (ii) within coding regions, and (iii) in UTRs (Supplemental Note S10).

Based on RNA sequencing of all samples, transcripts were predicted using our region-restricted probabilistic modeling method (RRPM, see Materials and Methods). Figure 2 gives an overview of features of the found transcripts. 3,331 genes that were not expressed or whose exon boundaries were not supported by the RNA-seq data were excluded from the 16,319 genes in the *S. commune* v3.0 reference genome. 15,522 transcripts were predicted for the remaining 12,988 genes. 10,703 genes (82%) had only one transcript, 85% of them being identical to the original annotation (Fig. 2a). A total number of 4,819 transcripts were found for the other 2,285 genes (see Fig. 2b). Most of these genes (90%) had two splice variants (Fig. 2b and Supplementary Note S3) and 72% of the alternative transcripts had only one alternative splicing event (Supplementary Note S4). The alternatively spliced genes are located over the entire genome (Supplementary Note S5). Together, 6,130 primitive splicing events resulted in the 4,819 transcripts of the 2,285 genes of which 65% (3,075) occur in the coding region of the gene, and 35% (2,155 genes) in the UTR (Fig. 2c).

Intron retention (33%) and alternative 5′ splicing site (25%) were the most common primitive events (Fig. 2f(i)). Alternative 3′ splicing sites and exon skipping events occurred at similar levels (21%). Mutually exclusive exons accounted for 93% of the 1,491 composite events, while multiple alternative 5′ and 3′ splicing sites were relatively rare at 4% and 3%, respectively (Fig. 2g(i)). These mutually exclusive exon events occurred in 171 genes representing 8% of the alternative spliced genes. Most alternative splicing events are not reading frame neutral (Supplementary Note S6).

UTRs of genes might stretch so far that they overlap with a neighboring gene. As a consequence, paired-end reads may lie within the boundary of another gene. To investigate whether such reads have a strong effect on event counts, we selected only those genes that are flanked on both sides by intergenic regions with zero coverage (i.e. no overlap). A total of 306 alternatively spliced genes were found in the selected set of 2,516 genes. We observed the same distribution of event counts in this set as compared to all genes (Supplementary Note S7). From this, we conclude that our analysis is not confounded by reads from neighboring genes.

### Alternative splicing is not limited to coding regions

Alternative splicing occurs not only in the translated regions of transcripts, but also in the UTRs of transcripts. RRPM is able to detect alternative splicing events that lie within the original gene boundaries of known genes. RRPM can detect alternative splicing events after premature stop codons introduced by alternative splicing events that still lie within the original gene boundaries (Supplementary Note S8). RRPM cannot investigate alternative splicing events in the UTRs extending outside of these original gene boundaries, and which are generally unknown. In the unknown UTR regions, we can use splice junction analysis (Supplementary Note S9).

A total of 3,975 (65%) alternative splicing events were found within coding transcript regions (Fig. 2c). Of these, 44% are intron retentions, and only 1% were exon skipping events (Fig. 2f(ii)). Due to splicing, some regions at the 5′ and 3′ ends of the original coding regions may become non-coding UTRs, and this is where the remaining 2,155 (35%) events are located. Of these, 1,225 (57%) were classified as exon skipping events and 293 (14%) as intron retentions (Fig. 2f(iii) and Supplementary Note S10). Mutually exclusive exons occur primarily in the untranslated regions of transcripts (Fig. 2g(ii,iii)). They account for 99% of all composite events in the UTR regions, but for only 15% of those in the coding regions. Multiple alternative 5′ and 3′ splicing sites, which are rare, occur mostly in coding regions.

Splice junction analysis enables a semi-quantitative analysis of alternative splicing in coding and non-coding regions (see Methods). We observed 186,221 splice junctions across all our RNA-seq samples. 29% of these occur only once, and are likely errors of the splicing machinery or sequencing. In the remaining 71%, we find 15,054 primary alternative splicing events. 62% lie within the coding regions of defined genes, and 38% are in the UTRs of these genes. We found 7,851 alternative (5′ or 3′) splicing sites and 1,527 exon skipping events inside coding regions, while we discovered 4,574 alternative splicing sites and 1,102 exon skipping events in UTRs. Alternative splicing is therefore clearly not limited to translated regions.

### Expression over developmental stages implicates alternative splicing in development

**Figure 3:**
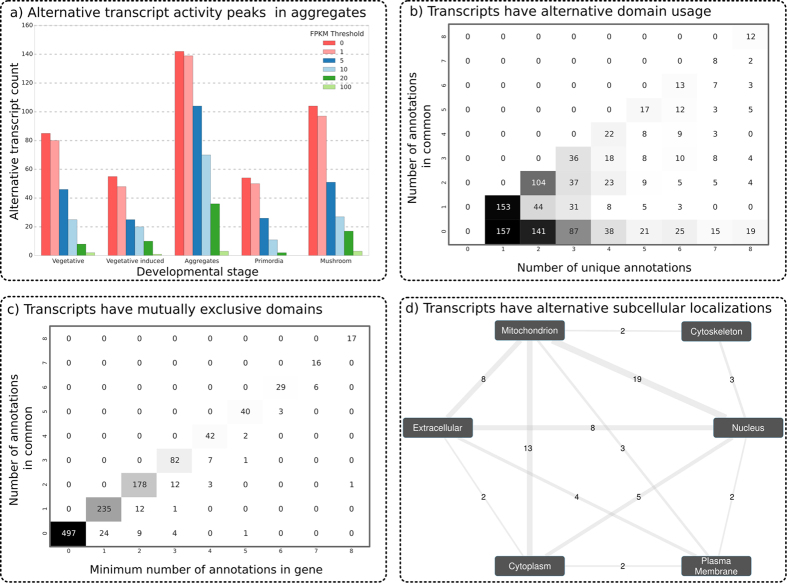
(**a**) Alternative splice variants of time course switch genes being more the abundant when compared to the annotated variant at different stages of activity of time course switch genes through development. We count the number of alternative transcripts which are active at each developmental stage, exceeding a particular expression threshold, and find that there are more active at the aggregates stage of development than at any other, regardless of expression threshold. (**b**) Implications of alternative splicing on domain predictions. The number of unique annotations are given on the x-axis, and the number of annotations in common between all transcripts is given on the y-axis. If the numbers are not equal, that means that not all transcripts in a gene are annotated with the same domains. In the corresponding cells are given the number of genes with this combination of annotations. Most genes do not exhibit alternative functionality (see diagonal), but quite a number do (see below the diagonal). (**c**) The smallest number of annotations of a transcript in a gene is given on the x-axis (e.g. a gene with two transcripts having 2 and 3 annotations would have a value of 2) and the number of annotations in common between all transcripts is given on the y-axis. On the diagonal are genes in which the number of annotations in common is limited by a transcript for which annotations are lost due to alternative splicing. Off the diagonal are genes that have enhanced their functional abilities through alternative splicing (see Supplemental Note S11). (**d**) Alternative splicing changes subcellular location of genes. Nodes represent a subcellular location. Edges represent genes that have two alternative splice variants, each encoding for a different subcellular location (connected nodes). Counts associated with an edge indicate the amount of genes having alternative transcripts that encode for the associated subcellular locations. For example, there are 8 genes for which the protein encoded by one transcript is secreted (extracellular) and the protein encoded by the other transcript is transported to the mitochondrion. See Supplemental Note S12 for genes whose subcellular location do not change.

**Figure 4:**
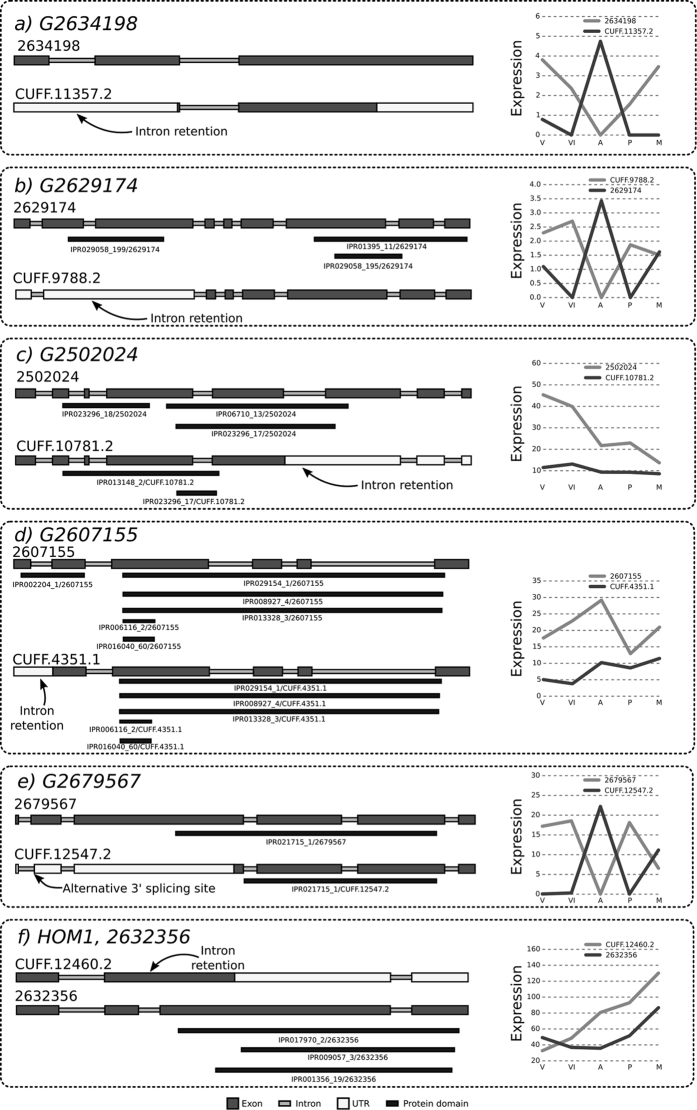
Structures of predicted alternative transcripts for selected genes. Genes are oriented from 5′ to 3′. Gray boxes are exons, white boxes are UTRs, thin light gray boxes are introns and dark gray boxes are protein domains. The primary (on average most expressed) transcript is always above the alternative transcript. The expression of the corresponding transcripts are shown on the left hand side of the figure, throughout the five developmental stages: Vegetative (V), Vegetative Induced (VI), Aggregates (A), Primordia (P) and, Mushroom (M). Refer to Supplemental File 1 for all transcript structures, and Supplemental Table 1 for expression and functional information for each transcript (**a**) Gene *G2634198*, encoding a carbohydrate active protein, with primary transcript 2634198 and secondary transcript CUFF.11357.2. An intron retention occurs in CUFF.11357.2 fusing the first two exons. (scaffold_9:981266–981925). (**b**) Gene *G2629174*, encoding a secreted protein. The second intron is retained. (scaffold_7:1,406,122–1,408,401). (**c**) Gene *G2502024*, encoding a carbohydrate active protein. The 5th intron is retained. (scaffold_6:428624–430098). (**d**) Gene *G2607155*, a metabolic gene. The first intron is retained. (scaffold_1:2733233–2735448). (**e**) Gene *G2679567*, a transcription factor. The secondary transcript has an A5SS on its second exon. (scaffold_8:1832047–1833917). (**f**) Gene *hom1*, (*G2632356*), a transcription factor involved in mushroom formation in *S. commune*. The primary transcript has a secondary transcript in which the second intron is retained. (scaffold_8:1757475–1758654).

Time Course Switching (TCS) genes differentially express splice variants during development^5^. The primary transcript (defined as the transcript that has the highest average expression over development), is replaced in expression by one of its alternative transcripts at some point in time. *S. commune* was found to have 425 of such genes, representing 865 transcripts. The aggregate stage contained more expressed alternative transcripts than any other stage (Fig. 3a). At this stage, the alternative transcripts of 142 genes were more abundant than their primary transcript. For example, the alternative transcript (ID CUFF.11357.2) of the carbohydrate active enzyme (cazyme) gene *G2634198* (Fig. 4a) entirely replaces the primary transcript (ID 2634198) during the aggregate stage, after which its abundance decreased to zero at the primordia stage, and in turn, replaced by the primary transcript. Gene *G2629174* (Fig. 4b) which encodes a secreted protein also exhibits this behavior. The polypeptide encoded by the alternative transcript lacks a large part of its N-terminus due to intron retention.

### Genes with alternative transcripts exhibit alternative functionality through protein domains

Alternative splicing may result in the gain or loss of functional domains. Out of the 2,285 genes with alternative transcripts, 1,257 (55%) have predicted functional domains. A functional domain is lost/gained in an alternative transcript in 897 (70%) genes (Fig. 3b,c and Supplementary Note S11). For 274 (12%) genes a domain is exchanged for another (Fig. 4c). For example, gene *G2502024*, encoding a predicted secreted cazyme, has two splice variants (Fig. 4c) both annotated with a glycosyl hydrolase domain (IPR domain IPR023296) involved in the hydrolysis of polysaccharide carbohydrates. The primary transcript (ID 2502024) is annotated with a glycoside hydrolase family 43 domain (IPR domain IPR006710), while the alternative transcript (ID CUFF.10781.2) has a different glycosyl hydrolase domain (IPR013148). The presence of alternative domain families in the two transcripts indicates alternative carbohydrate degradation abilities. 102 (24%) of the time course switching genes are predicted to have alternative functionality through alternative domain usage, implicating a change of function of those genes throughput development.

### Alternative splicing can affect the subcellular localization of encoded proteins

Alternative splicing may result in the gain or loss of a localization signal leading to a function in another compartment of the cell. For 936 alternatively spliced genes (41%) we were unable to predict a subcellular location for any transcripts (see Supplementary Note S12), and for 1278 genes (56%) we were unable to predict a subcellular location for all transcripts, but in 71 genes (3%), transcripts have different predicted localization signals (Fig. 3d).

Gene *G2607155* is an example of a predicted metabolic gene with two transcripts encoding proteins with alternative subcellular localizations (see Fig. 4d). The primary and alternative transcript, 2607155 and CUFF.4351.1, respectively, are both expressed. The protein encoded by the primary transcript is predicted to be transported to the mitochondrion, while the polypeptide encoded by the alternative transcript has an intron retention near the 5′ end of the transcript. As a result, it loses its first exon, destroying a valine catabolism-related3-hydroxyisobutyrate dehydrogenase site (IPR002204) specific to mitochondria, and the signaling peptides. The alternative transcript still retains the four deoxyhydrogenase (IPR029154, IPR008927, IPR013328, IPR006115) and the NADP binding domain (IPR016040) of the protein encoded by the primary transcript. Based on its remaining protein sequence, it is predicted that this secondary transcript is located in the cytoplasm, which indicates that alternative splicing may regulate the location of proteins in *S. commune*.

### Alternative splicing is present in key functional groups

Of particular interest are five gene functional groups: i) transcription factors, ii) cazymes, iii) secreted proteins, iv) cytochrome P450s, and v) metabolic genes. Alternative splicing occurs in all these groups (see Fig. 2e and Supplementary Note S13), and the set of cytochrome P450s are enriched in the set of alternatively spliced genes. The peak of alternative transcript expression in the aggregate stage persists across all these functional groups regardless of expression threshold, demonstrating that alternative splicing is actively regulated in these functional groups (see Supplementary Note S14).

Alternative splicing in a transcription factor gene may impact a DNA binding domain or a protein interaction domain. As an example, the alternative transcript of gene *G2679567* has an alternative 5′ splicing site in its second exon (see Fig. 4e) resulting in a shortened DNA binding domain (IPR021715) due to the loss of most of the third exon. This alternative transcript is differentially expressed across developmental stages, suggesting that the regulation program also changes with alternative splicing. Genes encoding secreted proteins that are alternatively spliced may impact the signal sequence for secretion located in the N-terminal of the protein. The alternative transcript of the TCS gene *G2629174* is the result of an intron retention (see Fig. 4b). As a result, the N-terminal signal sequence is affected, which is predicted to prevent the protein from being be secreted. If this is the case then, based on the expression of these transcripts, this protein is secreted at different rates throughout development. Many secreted proteins may take on alternative locations in the cell (Fig. 3d). In 22 cases, secreted proteins (in the extracellular compartment) are predicted to end up in other subcellular locations (8 in the mitochondrion, 8 in the nucleus, 4 in the plasma membrane and 2 in the cytoplasm), and in 62 cases, we were unable to predict the subcellular location (Supplementary Note S12).

### Implications on previously studied genes of *S. commune*

Only 84 *S. commune* genes have been assigned names, 13 of them have alternative splicing variants (see Supplementary Note S15). Among these genes are the mating type genes *aay4, abu6*, and *bbp2*–*1*, transcription factor genes *crea* and HOM1 that encode the carbon catabolite repression regulator, and the fruiting transcription factor Hom1. The alternative transcripts in the mating type genes have no effect on function. In contrast, alternative splicing impacts *hom1*. Its primary transcript retains the second intron (see Fig. 4f). This produces a frame shift that prematurely ends the coding region. Due to this, it loses its homeobox domain and thus will not function as a transcription factor. Notably, this *hom1* transcript is the most abundant throughout development.

## Discussion

Previous studies characterizing alternative splicing in fungi unequivocally found intron retentions to be the most common events^10^,^11^,^12^. This study confirmed this finding with intron retentions accounting for 33% of the events. Notably, they are enriched in coding regions, where they account for 43% of the events, while exon skipping occurred primarily in UTRs. With 1% of the events, exon skipping was rare in coding regions, but they represented 57% of the events in UTRs. The prevalence of intron retention contrasts the dominance of exon skipping in mammals^19^,^20^. Our results strongly suggest that fungi use alternative splicing events differently when compared to mammalian genomes. Based on event prevalence they are more similar to plants, where intron retentions are also the most common event^21^.

Most alternative splicing events (64%) were not reading frame neutral, indicating that there is no strong selective pressure for reading frame neutral alternative splicing events. This is also in contrast with alternative splicing in mammals, where there appears to be a selection for reading-frame neutral events^19^,^20^.

The incidence of alternative splicing was much higher compared to previous studies. Previous studies examined primarily ascomycetes and few basidiomycetes, only two of which form mushrooms^9^,^10^. This may imply that more developmentally complex fungal species have more alternative splicing (see Fig. 1e and Supplementary Note S1) with basidiomycetes having the most alternative splicing, followed by pathogenic ascomycetes, then plant pathogenic ascomycetes and finally, saprobic ascomycetes and yeasts.

The genome of *S. commune* encodes 16,319 genes. The median intergenic distance is 539 bp, making the gene density very high when compared to plants and humans^22^,^23^. This hampers studying alternative splicing in *S. commune*, and therefore, we focused only on annotated genes. This prevents the discovery of splicing events at novel loci but allowed us to identify alternatively spliced transcripts of the annotated genes and to study the functional impact of these splicing events. Recent studies from the ENCODE project^23^ have shown that significant portions of non-coding regions of the human genome are expressed as mRNAs, and are spliced. By analyzing splice junctions, we observed 5,676 alternative splicing events in non-coding regions, demonstrating that *S. commune* also makes use of alternative splicing in non-coding regions. Due to the density of the genome, events that lie in UTRs cannot be associated with a gene using short read data. Use of long read technologies will alleviate technological problems in assembling the splice variants as those reads will cover entire transcripts.

In predicting the protein sequences of our transcripts, we assumed that the longest Open Reading Frame (ORF) is the one that is most likely to be translated. This is a common assumption in gene prediction software, yet, as a consequence, different transcripts within a gene may have different start codons. The Kozak consensus sequence is a good indicator of translation initiation^24^. The coding sequences predicted in our alternative transcripts indeed are supported by Kozak consensus sequences (see Supplement Note S17). But, translation initiation is still poorly understood and predicting exactly where translation should initiate (i.e. which ORF is actually translated) is still difficult^25^. It has been shown that there are alternative AUG start codons which produce different proteins from the same transcript^26^,^27^. Even beyond that, there are ORFs in the upstream UTRs that are also translated^28^. Future studies of alternative splicing should extend their perspective from the current “one gene, many transcripts”, to the “one transcript many proteins” viewpoint.

Finally, we should note that we have been conservative in our prediction of alternative transcripts and alternative splicing events. Additionally, although we sampled across the whole development of *S. commune*, all samples were grown under the same environmental conditions (see Methods) in an isogenic strain (see Supplementary Note S16). Hence, alternative splicing may be much more abundant and provide much more functionality than we observe in this study.

## Conclusions

Alternative splicing was found to increase proteome complexity in *S. commune* by the gain or loss of functional domains or localization signals. Alternative splicing was found in 18% of expressed genes, resulting in 20% more transcripts. Yet, the real incidence of alternative splicing is probably higher. More than two thirds of the alternatively spliced genes exhibited alternative functionality by coding for different domains or by acquiring alternative subcellular localizations. Selected functional groups were shown to contain alternatively spliced genes, with the cytochrome P450 genes showing the highest proportion. Time Course Switching genes that differentially express gene variants throughout development represented 19% of the alternatively spliced genes. Of these genes, 24% were predicted to have alternative functionality. We thus show that alternative splicing dynamically varies the complexity of the proteome encoded in the dense genome of *S. commune*.

It has long been believed that alternative splicing does not contribute functionality to the cell in plants, or fungi, despite the presence of alternative splicing events^29^. Alternative splicing events were generally thought to result in non-functional proteins or to be similar to other transcripts of the same gene, resulting in retained functionality. Similarly, in the early stages of the ENCODE project it was also commonly believed that the vast majority of alternative mammalian transcripts do not have functional roles^30^. Now, the role of alternative splicing is commonly accepted in humans^31^ and its tremendous potential influence on function is being accepted in plants^32^,^33^,^34^. With this work, we extend this notion to fungi.

## Methods

### *S. commune* genome and annotations

The genome sequence of *S. commune* v3.0 was retrieved from the Joint Genome Initiative database^35^, together with gene definitions (GFF), Interpro and GO annotations.

### mRNA isolation

mRNA was isolated from *S. commune* strain H4–8^36^ grown at 25 °C on minimal medium containing 1% glucose and 1.5% agar^37^. RNA was sampled during (i) vegetative growth, (ii) induced vegetative growth, (iii) aggregate formation,(iv) primordia formation, and (v) when mushroom had developed. In the induced vegetative growth stage, the colony has been exposed to light for some time but has not yet started forming aggregates. RNA was also isolated from 9 dikaryotic deletion strains at two time points^16^,^17^,^18^: Δ*wc*-*1*Δ*wc*-*1*; Δ*wc*-*2*Δ*wc*-*2*; Δ*hom1*Δ*hom1*; Δ*hom2*Δ*hom2*; Δ*fst3*Δ*fst3*; Δ*fst4*Δ*fst4*; Δ*bri1*Δ*bri1*; Δ*gat1*Δ*gat1*; and Δ*c2h2*Δ*c2h2*. The sample at the first time point was taken at the time where a simultaneously growing wildtype sample reached the aggregates stage of development. The sample at the second time point was taken when the wildtype had formed mature mushrooms (see Supplementary Note S18).

### RNA sequencing

Samples were sequenced with 100 bp paired-end sequencing on the HiSeq 2000 Illumina platform with an average of 1.7 Gb of reads per sample (average 43x genome coverage). Exceptions are the wildtype stages i) vegetative growth, ii) induced vegetative growth and v) primordia, which were sequenced at a later date with 125 bp paired end sequences on the HiSeq 2500 platform with an average of 5.0 Gb of reads per sample. All samples were produced in duplo for use in transcript abundance estimation. Raw RNA-seq reads are filtered for quality using trimmomatic^38^. These RNA-seq samples have been made available under BioProject PRJNA323434.

### Region-restricted probabilistic modeling (RRPM) for alternative transcript discovery

Due to the high gene-density of *S. commune*, very few neighboring genes have intergenic regions without expression. In order to reduce the interference of neighboring UTRs, we restricted the discovery of alternative transcripts to gene coding regions only. To this end, the genome was split at gene boundaries into as many fragments as there are genes, each fragment representing a different annotated gene region on the *S. commune* genome.

Reads from all samples were aligned to the collection of gene-based fragments using STAR^39^, producing a splice junction database used to optimize STAR alignments in a second alignment round (we use all samples to have a comprehensive database). The second round of STAR produced BAM files sorted by coordinate. Reads not aligning entirely within a gene-based fragment were discarded. Using these BAM files, Cufflinks^14^ was run in Reference Annotation Based Transcript Assembly (RABT) mode, to produce a set of predicted transcripts. Although the transcripts were predicted on the basis of all samples, it does not mean that these transcripts are also active in every sample. These transcripts were then projected back onto the original genome to restore their context. See Supplementary Note S19 for a schematic of RRPM, and Supplementary Note S20 for parameter settings used for the tools mentioned above.

### Transcript filtering

The output from Cufflinks was filtered to remove transcripts resulting from noise: 1) predicted transcripts which do not lie on the same strand as the original gene description were removed (they cannot be accurately called without strand-specific reads); 2) transcripts which had no expression or did not have reads supporting its splice junctions were removed; 3) transcripts which did not contain Open Reading Frames (ORFs) of at least length 40 nt were removed. See Supplementary Note S21 for an overview of how many genes and transcripts were filtered at each step. The final output is a list of genes with their corresponding transcripts in GFF format.

### Calling alternative splicing events

To identify alternative splicing events, we must compare the found transcripts against a reference. Comparing transcripts to the original reference annotation may result in peculiarities due to a lagging quality of annotation. For example, the original annotation may describe a large exon, whereas all predicted transcripts indicate only two smaller exons. To find a suitable reference, we established a consensus annotation based on all expressed transcripts for that gene. The consensus annotation contains all distinctly observed exons in a gene, without intron retentions (see Supplementary Note S22). Consequently, the consensus annotation is not necessarily one of the transcripts of the gene. In fact, it might not even contain a valid ORF. It just serves as a reference to describe observed splice variations.

We called primary splice events by′ aligning the exons of an observed transcript to the consensus annotation. From this alignment we determine the absence of an exon (ES), the fusion of exons (IR), or the extension or contraction of exon boundaries (A5SS, A3SS) (see Supplementary Note S23). Composite events “multiple alternative 5′ and “multiple alternative 3′ splicing sites are classified by grouping exons of transcripts within one gene and counting the number of alternative exon boundaries for each exon. Mutually exclusive exons were detected by counting how often pairs of exons occur across all transcripts. If a pair was never observed across all transcripts in a gene but individually the exons are observed, then we called that pair a mutually exclusive exon event.

We counted the number of alternative splicing events at three levels. At the event level, we counted each alternative splicing event. If, for example, three transcripts exist in a gene, and in two of these transcripts the third exon is skipped, we count this as two events. At the transcript level, we counted each transcript with a particular event. Thus, when a transcript skips two exons, we count it only once. At the gene level, we count the number of genes with a specific alternative splicing event. For example, when a gene has two transcripts that each skip different exons we would counted that as one event.

### Detection of untranslated splicing events

We quantify splicing events outside of the translated regions similar to Xie *et al*.^12^ through the study of clusters of splice junctions^12^. The slight differences are described in Supplementary Note S9.

### Estimating transcript expression profile of development

Abundance estimates for each transcript were computed by the Cufflinks suite. For each sample separately, we aligned both replicates to the entire genome with STAR in the same two-round procedure as used for transcript prediction. We provided the resulting BAM files of both replicates to Cufflinks, which estimates the abundances of the transcripts in both replicated samples together. Doing this for each developmental stage results in an expression profile for each predicted transcript.

### Time Course Switches

Based on the expression profiles of each transcript, we detected time course switching genes for each gene using the method described by Lees *et al*.^5^. Briefly, we compare the expression of the primary transcript (which has the highest average expression across time) as well as the expression of an alternative transcript. Intuitively speaking, this comparison scores the relative expression of both transcripts in such a way that the score is larger when the expression profiles of the two transcripts are more dissimilar in shape. As in Lees *et al*.^5^, genes which have a score greater than 0.5 are considered to be time course switches. This results in a list of genes which have alternative transcripts that swap expression with another.

### Functional annotations and group definitions

#### Functional domain annotations

InterPro domain annotations were predicted for the translated protein sequence of the longest ORF in each predicted transcript using InterProScan^40^. We re-index these annotations based on location. If a gene has the same annotation twice, but in two different locations, we assign each of them new identifiers. This allows us to more specifically identify alternative functionality by domain annotations.

#### Transcription factor definitions

Transcription factors were predicted based on 83 InterProDNA-binding or regulatory domains (see Supplementary Note S24) suggested by the Fungal Transcription Factor Database^41^,^42^. Transcription factors are predicted for the original gene definitions. If one of these InterPro domains was present in the protein sequence of a gene, then the gene was called as a transcription factor.

#### Carbohydrate-active enzymes prediction

Using the Cazymes Analysis Toolkit (CAT)^43^, we predicted carbohydrate-active enzymes based on the original gene definitions. If a gene’s protein sequence was predicted to be a cazyme by either the sequence-based annotation method or the Pfam-based annotation method then we considered it a cazyme.

#### Secreted Proteins prediction

We used the same procedure as^44^ to predict secreted proteins. Briefly, genes with SignalP^45^ signal peptides, or a TargetP^46^ Loc = S were kept. The remaining genes were further filtered with TMHMM^47^, keeping only genes with zero or one transmembrane domains. Finally, genes were filtered using Wolf PSort^48^ to select genes with a Wolf PSort extracellular score greater than 17.

#### Metabolic and Cytochrome P450 gene groups

Genes with the GO annotation “metabolic process” (annotation ID: GO:0008152) were called as metabolism genes. Genes with the IPR annotation IPR001128 were used as Cytochrome P450 genes.

#### Subcellular location prediction

We used Wolf PSort^48^ to predict the subcellular localization of alternatively spliced transcripts. Protein sequences for each transcript were constructed, and provided to Wolf PSort. All annotations with a score below 17 were removed^44^.

## Additional Information

**Accession codes:** The RNA-seq data and relevant metadata can be found under BioProject PRJNA323434.

**How to cite this article**: Gehrmann, T. *et al. Schizophyllum commune* has an extensive and functional alternative splicing repertoire. *Sci. Rep.* **6**, 33640; doi: 10.1038/srep33640 (2016).

## Supplementary Material

Supplementary Information

Supplementary Dataset 1

Supplementary Dataset 2

## References

[b1] Medina M. W. & Krauss R. M. Alternative splicing in the regulation of cholesterol homeostasis. Current Opinion in Lipidology 24, 147–152, URL http://content.wkhealth.com/linkback/openurl?sid=WKPTLP:landingpage&an=00041433-201304000-00009 (2013). 23314925 10.1097/MOL.0b013e32835cf284 PMC3667406

[b2] Oltean S. & Bates D. O. Hallmarks of alternative splicing in cancer. Oncogene 33, 1–8, URL http://www.ncbi.nlm.nih.gov/pubmed/24336324 (2013). 24336324 10.1038/onc.2013.533

[b3] Gueroussov S. . An alternative splicing event amplifies evolutionary differences between vertebrates. Science 349, 868–873, URL http://www.sciencemag.org/cgi/doi/10.1126/science.aaa8381 (2015). 26293963 10.1126/science.aaa8381

[b4] Kwon Y.-J., Park M.-J., Kim S.-G., Baldwin I. T. & Park C.-M. Alternative splicing and nonsense-mediated decay of circadian clock genes under environmental stress conditions in Arabidopsis. BMC Plant Biology 14, 136, URL http://www.biomedcentral.com/1471-2229/14/136 (2014). 24885185 10.1186/1471-2229-14-136 PMC4035800

[b5] Lees J. G., Ranea J. A. & Orengo C. A. Identifying and characterising key alternative splicing events in Drosophila development. BMC Genomics 16, 608, URL http://dx.doi.org/10.1186/s12864-015-1674-2 http://www.biomedcentral.com/1471-2164/16/608 (2015). 26275604 10.1186/s12864-015-1674-2 PMC4537583

[b6] Vilardell J., Chartrand P., Singer R. H. & Warner J. R. The odyssey of a regulated transcript. RNA (New York, NY) 6, 1773–1780 (2000). 10.1017/s135583820000145x PMC1370047 11142377

[b7] Boel E., Hjort I., Svensson B., Norris F. & Fiil N. P. Glucoamylases G1 and G2 from Aspergillus niger are synthesized from two different but closely related mRNAs. The EMBO journal 3, 1097–1102, URL http://www.ncbi.nlm.nih.gov/pmc/articles/PMC557479/ (1984). 6203744 10.1002/j.1460-2075.1984.tb01935.x PMC557479

[b8] Okazaki K. & Niwa O. mRNAs encoding zinc finger protein isoforms are expressed by alternative splicing of an in-frame intron in fission yeast. DNA research: an international journal for rapid publication of reports on genes and genomes 7, 27–30 (2000). 10718196 10.1093/dnares/7.1.27

[b9] Grützmann K. . Fungal alternative splicing is associated with multicellular complexity and virulence: A genome-wide multi-species study. DNA Research 21, 27–39 (2014). 24122896 10.1093/dnares/dst038 PMC3925392

[b10] McGuire A. M., Pearson M. D., Neafsey D. E. & Galagan J. E. Cross-kingdom patterns of alternative splicing and splice recognition. Genome biology 9, R50, URL http://www.pubmedcentral.nih.gov/articlerender.fcgi?artid=2397502&tool=pmcentrez&rendertype=abstract (2008). 18321378 10.1186/gb-2008-9-3-r50 PMC2397502

[b11] Zhao C., Waalwijk C., de Wit P. J. G. M., Tang D. & van der Lee T. RNA-Seq analysis reveals new gene models and alternative splicing in the fungal pathogen Fusarium graminearum. BMC genomics 14, 21, URL http://www.pubmedcentral.nih.gov/articlerender.fcgi?artid=3577648&tool=pmcentrez&rendertype=abstract (2013). 23324402 10.1186/1471-2164-14-21 PMC3577648

[b12] Xie B.-B. . Deep RNA sequencing reveals a high frequency of alternative splicing events in the fungus Trichoderma longibrachiatum. BMC genomics 16, 54, URL http://www.ncbi.nlm.nih.gov/pubmed/25652134 (2015). 25652134 10.1186/s12864-015-1251-8 PMC4324775

[b13] Grabherr M. G. . Full-length transcriptome assembly from RNA-Seq data without a reference genome. Nature biotechnology 29, 644–652, URL http://www.pubmedcentral.nih.gov/articlerender.fcgi?artid=3571712&tool=pmcentrez&rendertype=abstract (2011). 10.1038/nbt.1883 PMC3571712 21572440

[b14] Trapnell C. . Transcript assembly and quantification by RNA-Seq reveals unannotated transcripts and isoform switching during cell differentiation. Nature biotechnology 28, 511–515 , URL http://www.pubmedcentral.nih.gov/articlerender.fcgi?artid=3146043&tool=pmcentrez&rendertype=abstract (2010). 10.1038/nbt.1621 PMC3146043 20436464

[b15] Gordon S. P. . Widespread polycistronic transcripts in fungi revealed by single-molecule mRNA sequencing. Plos One 10, 1–15, URL http://dx.doi.org/10.1371/journal.pone.0132628 (2015). 10.1371/journal.pone.0132628 PMC4503453 26177194

[b16] Ohm R. a., Aerts D., Wösten H. a. B. & Lugones L. G. The blue light receptor complex WC-1/2 of Schizophyllum commune is involved in mushroom formation and protection against phototoxicity. Environmental Microbiology 15, 943–955, URL http://www.ncbi.nlm.nih.gov/pubmed/22998561 (2013). 22998561 10.1111/j.1462-2920.2012.02878.x

[b17] Ohm R. a. . An efficient gene deletion procedure for the mushroom-forming basidiomycete Schizophyllum commune. World journal of microbiology & biotechnology 26, 1919–1923, URL http://www.pubmedcentral.nih.gov/articlerender.fcgi?artid=2940052&tool=pmcentrez&rendertype=abstract (2010). 20930926 10.1007/s11274-010-0356-0 PMC2940052

[b18] Ohm R. a., de Jong J. F., de Bekker C., Wösten H. a. B. & Lugones L. G. Transcription factor genes of Schizophyllum commune involved in regulation of mushroom formation. Molecular Microbiology 81, 1433–1445 (2011). 21815946 10.1111/j.1365-2958.2011.07776.x

[b19] Sammeth M., Foissac S. & Guigó R. A general definition and nomenclature for alternative splicing events. Plos Computational Biology 4 (2008). 10.1371/journal.pcbi.1000147 PMC2467475 18688268

[b20] Xing Y. & Lee C. Alternative splicing and RNA selection pressure–evolutionary consequences for eukaryotic genomes. Nature reviews. Genetics 7, 499–509 (2006). 10.1038/nrg1896 16770337

[b21] Chamala S., Feng G., Chavarro C. & Barbazuk W. B. Genome-Wide Identification of Evolutionarily Conserved Alternative Splicing Events in Flowering Plants. Frontiers in Bioengineering and Biotechnology 3, URL http://www.frontiersin.org/Bioinformatics_and_Computational_Biology/10.3389/fbioe.2015.00033/abstract (2015). 10.3389/fbioe.2015.00033 PMC4374538 25859541

[b22] Alexandrov N. N. . Features of Arabidopsis genes and genome discovered using full-length cDNAs. Plant Molecular Biology 60, 69–85 (2006). 16463100 10.1007/s11103-005-2564-9

[b23] Djebali S. . Landscape of transcription in human cells. Nature 489, 101–108, URL http://www.pubmedcentral.nih.gov/articlerender.fcgi?artid=3684276&tool=pmcentrez&rendertype=abstract (2012). 22955620 10.1038/nature11233 PMC3684276

[b24] Kozak M. An analysis of 5′-noncoding sequences from 699 vertebrate messenger RNAs. Nucleic acids research 15, 8125–48, URL http://www.pubmedcentral.nih.gov/articlerender.fcgi?artid=306349&tool=pmcentrez&rendertype=abstract (1987). 3313277 10.1093/nar/15.20.8125 PMC306349

[b25] Zur H. & Tuller T. New universal rules of eukaryotic translation initiation fidelity. Plos computational biology 9, e1003136, URL http://www.pubmedcentral.nih.gov/articlerender.fcgi?artid=3708879&tool=pmcentrez&rendertype=abstract (2013). 23874179 10.1371/journal.pcbi.1003136 PMC3708879

[b26] Ingolia N. T. Ribosome profiling: new views of translation, from single codons to genome scale. Nature reviews. Genetics 15, 205–13, URL http://www.ncbi.nlm.nih.gov/pubmed/24468696 (2014). 10.1038/nrg3645 24468696

[b27] Kochetov A. V. Alternative translation start sites and hidden coding potential of eukaryotic mRNAs. Bio Essays 30, 683–691 (2008). 10.1002/bies.20771 18536038

[b28] Starck S. R. . Translation from the 5′ untranslated region shapes the integrated stress response. Science 351, aad3867–aad3867, URL http://www.sciencemag.org/cgi/doi/10.1126/science.aad3867 (2016). 26823435 10.1126/science.aad3867 PMC4882168

[b29] Syed N. H., Kalyna M., Marquez Y., Barta A. & Brown J. W. S. Alternative splicing in plants - coming of age. Trends in Plant Science 17, 616–623, URL http://dx.doi.org/10.1016/j.tplants.2012.06.001 (2012). 22743067 10.1016/j.tplants.2012.06.001 PMC3466422

[b30] Tress M. L. . The implications of alternative splicing in the ENCODE protein complement. Proceedings of the National Academy of Sciences of the United States of America 104, 5495–5500 (2007). 17372197 10.1073/pnas.0700800104 PMC1838448

[b31] Keren H., Lev-Maor G. & Ast G. Alternative splicing and evolution: diversification, exon definition and function. Nature reviews. Genetics 11, 345–355, URL http://dx.doi.org/10.1038/nrg2776 (2010). 10.1038/nrg2776 20376054

[b32] Barbazuk W. B., Fu Y. & McGinnis K. M. Genome-wide analyses of alternative splicing in plants: Opportunities and challenges. Genome Research 18, 1381–1392 (2008). 18669480 10.1101/gr.053678.106

[b33] Reddy A. S. N., Marquez Y., Kalyna M. & Barta A. Complexity of the Alternative Splicing Landscape in Plants. The Plant Cell 25, 3657–3683, URL http://www.plantcell.org/cgi/doi/10.1105/tpc.113.117523 (2013). 24179125 10.1105/tpc.113.117523 PMC3877793

[b34] Filichkin S., Priest H. D., Megraw M. & Mockler T. C. Alternative splicing in plants: directing traffic at the crossroads of adaptation and environmental stress. Current Opinion in Plant Biology 24, 125–135, URL http://linkinghub.elsevier.com/retrieve/pii/S136952661500028X (2015). 25835141 10.1016/j.pbi.2015.02.008

[b35] Grigoriev I. V. . The genome portal of the Department of Energy Joint Genome Institute. Nucleic acids research 40, D26–32, URL http://www.pubmedcentral.nih.gov/articlerender.fcgi?artid=3245080&tool=pmcentrez&rendertype=abstract (2012). 22110030 10.1093/nar/gkr947 PMC3245080

[b36] Ohm R. a. . Genome sequence of the model mushroom Schizophyllum commune. Nature biotechnology 28, 957–63, URL http://www.ncbi.nlm.nih.gov/pubmed/20622885 (2010). 10.1038/nbt.1643 20622885

[b37] Van Peer A. F., De Bekker C., Vinck A., Wösten H. a. B. & Lugones L. G. Phleomycin increases transformation efficiency and promotes single integrations in schizophyllum commune. Applied and Environmental Microbiology 75, 1243–1247 (2009). 19114524 10.1128/AEM.02162-08 PMC2648172

[b38] Bolger A. M., Lohse M. & Usadel B. Trimmomatic: A flexible trimmer for Illumina sequence data. Bioinformatics 30, 2114–2120 (2014). 24695404 10.1093/bioinformatics/btu170 PMC4103590

[b39] Dobin A. . STAR: ultrafast universal RNA-seq aligner. Bioinformatics (Oxford, England) 29, 15–21, URL http://www.ncbi.nlm.nih.gov/pubmed/23104886 (2013). 10.1093/bioinformatics/bts635 PMC3530905 23104886

[b40] Zdobnov E. M. & Apweiler R. InterProScan–an integration platform for the signature-recognition methods in InterPro. Bioinformatics (Oxford, England) 17, 847–848 (2001). 10.1093/bioinformatics/17.9.847 11590104

[b41] Park J. . CFGP: A web-based, comparative fungal genomics platform. Nucleic Acids Research 36, D562–571, URL http://www.pubmedcentral.nih.gov/articlerender.fcgi?artid=2238957&tool=pmcentrez&rendertype=abstract (2008). 17947331 10.1093/nar/gkm758 PMC2238957

[b42] Park J. . FTFD: an informatics pipeline supporting phylogenomic analysis of fungal transcription factors. Bioinformatics (Oxford, England) 24, 1024–1025, URL http://www.ncbi.nlm.nih.gov/pubmed/18304934 (2008). 10.1093/bioinformatics/btn058 18304934

[b43] Lombard V., Golaconda Ramulu H., Drula E., Coutinho P. M. & Henrissat B. The carbohydrate-active enzymes database (CAZy) in 2013. Nucleic Acids Research 42, D490–D495, URL http://nar.oxfordjournals.org/lookup/doi/10.1093/nar/gkt1178 (2014). 24270786 10.1093/nar/gkt1178 PMC3965031

[b44] Morais do Amaral A., Antoniw J., Rudd J. J. & Hammond-Kosack K. E. Defining the Predicted Protein Secretome of the Fungal Wheat Leaf Pathogen Mycosphaerella graminicola. Plos One 7, 1–19 (2012). 10.1371/journal.pone.0049904 PMC3517617 23236356

[b45] Petersen T. N., Brunak S. r., von Heijne G. & Nielsen H. SignalP 4.0: discriminating signal peptides from transmembrane regions. Nature Methods 8, 785–786, URL http://www.nature.com/doifinder/10.1038/nmeth.1701 (2011). 21959131 10.1038/nmeth.1701

[b46] Emanuelsson O. & Nielsen H. Predicting subcellular localization of proteins based on their N-terminal amino acid sequence. Journal of Molecular Biology 300, 1005–1016, URL http://www.sciencedirect.com/science/article/pii/S0022283600939032 (2000). 10891285 10.1006/jmbi.2000.3903

[b47] Krogh A., Larsson B., von Heijne G. & Sonnhammer E. L. Predicting transmembrane protein topology with a hidden markov model: application to complete genomes. Journal of Molecular Biology 305, 567–580, URL http://linkinghub.elsevier.com/retrieve/pii/S0022283600943158 (2001). 11152613 10.1006/jmbi.2000.4315

[b48] Horton P. . WoLF PSORT: protein localization predictor. Nucleic Acids Research 35, W585–W587, URL http://nar.oxfordjournals.org/lookup/doi/10.1093/nar/gkm259 (2007). 17517783 10.1093/nar/gkm259 PMC1933216

