# Supplementary material for: Schizophyllum commune has an extensive and functional alternative splicing repertoire
Source: Sci Rep. 2016 Sep 23;6:33640. doi: 10.1038/srep33640 (PMC5034255; doi:10.1038/srep33640)
Supplement: Supplementary Information [file srep33640-s1.pdf]

Supplementary Notes for

Schizophyllum commune has an extensive and functional alternative splicing repertoire

Thies Gehrmann, Jordi F. Pelkmans, Luis G. Lugones, Han A.B. Wösten, Thomas Abeel, Marcel J. T. Reinders

Note S1) Relating to Background: Phylogeny and complexity of species indicates use of alternative splicing

We constructed a phylogenetic tree for the fungal species studied in (McGuire et al., 2008), (Zhao et al., 2013) as well as *Schizophyllum commune*. We did not include (Grützmann et al., 2014) as they did not report the number of alternatively spliced variants per genome. The phylogenetic tree was constructed with PhyloT (http://phylot.biobyte.de/) based on the species and the taxonomic IDs given in ST1.1 and visualized with the Interactive Tree Of Life (Letunic and Bork, 2011). We augmented the phylogenetic tree (shown in Figure 1 of the main text) with the number of alternatively spliced transcripts per species. The occurrence of alternative splicing increases with species complexity. The basidiomycetes have the most alternatively spliced transcripts, followed by pathogenic ascomycetes, plant pathogenic ascomycetes and, finally, filamentous ascomycetes and yeasts. The number of alternatively spliced transcripts is, however, not consistent across the different clades of the tree, indicating that the alternative splicing machinery is common to all fungi, but increases and decreases in each clade depending upon species specialization.

Some care must be taken to interpret the numbers from (McGuire et al., 2008) as they are based on incomplete EST databases, and the number of spliced ESTs is highly correlated with the number of alternatively spliced transcripts (r=0.949, see Figure SF1.1), indicating a dependence upon a high quality EST dataset.

Table ST1.1: The species names, splice variants, and taxonomic IDs used to construct the phylogenetic tree in Figure SF1.1.

Species	Number of splice variants	NCBI Taxonomy ID
S. pombe	3	4896
S. cerevisiae	9	4932
N. crassa	20	5141
P. nodorum	36	420364
R. oryzae	47	64495
H. capsulatum	50	5037
U. maydis	85	5270
A. nidulans	100	162425
M. grisea	151	148305
A. flavus	162	5059
F. graminarium	231	5518
S. sclerotiorum	323	5180
C. immitis	861	5501
C. posadasii	950	199306
C. neoformans	1091	5207
S. commune	4819	5334

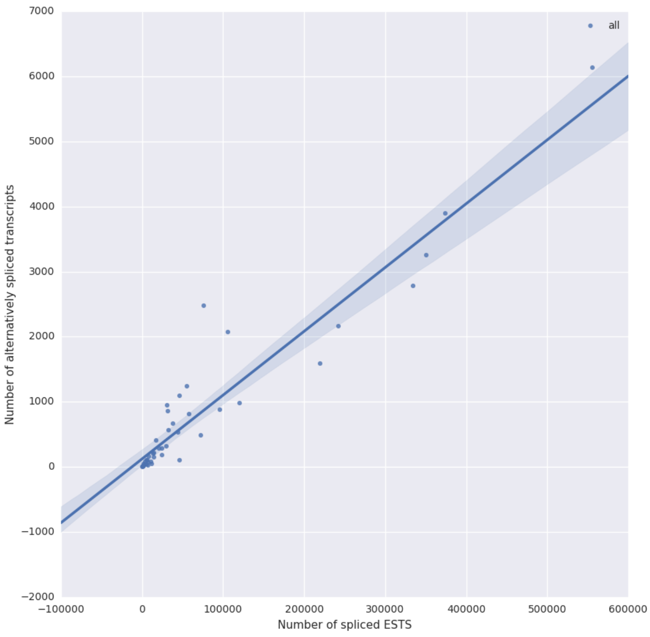

Figure SF1.1: Correlation (r=0.949) between the number of spliced ESTs and the number of detected alternatively spliced transcripts in the species studied by McGuire et. al. (McGuire et al., 2008).

Note S2) Relating to Background: The gene-density of S. commune

As many fungal genomes, the *S. commune* genome is very gene-dense. The median distance between coding sequences is only 539 nucleotides. The intergenic regions are even shorter due to the UTRs. For comparison: the recent ENCODE studies (Djebali et al., 2012) reported a median intergenic region of about 4,000 bp for the human genome.

In *S. commune*, 13.5% of the genome is intronic, and 52.7% of the genome is predicted to be coding. The remaining 33.8% are untranslated regions or intergenic, non-coding regions. Based on our RNA-Seq data, 88.6% of the genome is expressed. This means that significant portions of the non-coding and intronic sequences are expressed. This makes it very common for neighboring gene transcripts to overlap with each other.

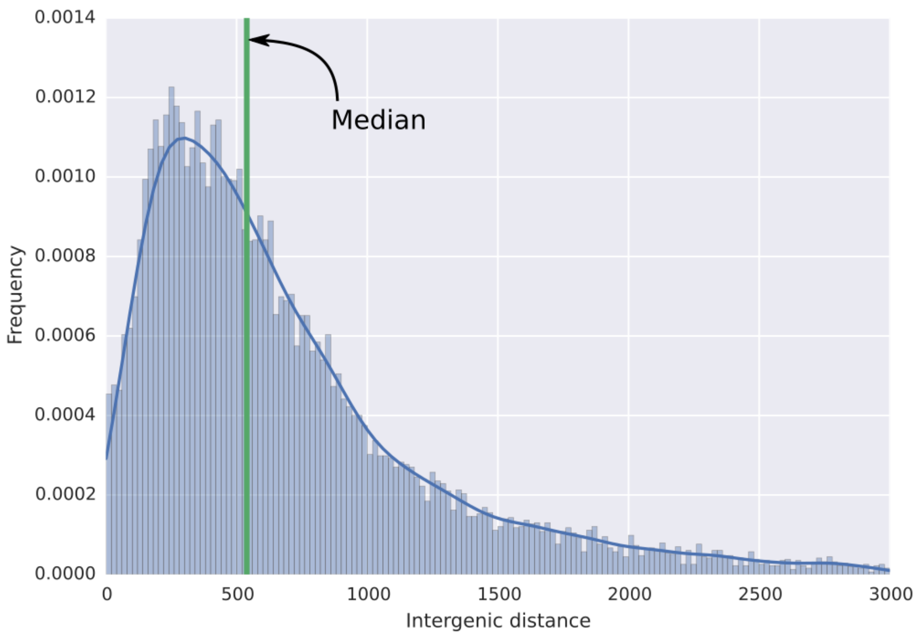

Figure SF2.1: The distribution of intergenic distances in S. commune. The median is indicated by a green line.

Note S3) Relating to figure 2B: The number of genes with 1 or more transcripts

Most expressed genes have only 1 alternative transcript, but many have more than that. Even then, 90% of all genes which have alternative splicing have two alternative splicing variants. Table ST3.1 shows the distribution of the number of transcripts per gene.

Table ST3.1: The distribution of alternative transcripts over genes. Most genes have only one transcript.

Number of transcripts	Number of genes
1	10,703
2	2,058
3	208
4	16
5	3

Note S4) Relating to Results: Alternative splicing events distribution per transcript

Relative to the consensus structure generated from the number of references, transcripts may have more than one alternative splicing event occurring on the transcript. Table ST4.1 shows the distribution of the number of alternative splicing events per transcript.

Table ST4.1: The number of transcripts with a certain number of events.

Number of events	Number of transcripts
1	2,787
2	808
3	127
4	58
5	26
6	16
7	12
8	5
9	12
10	4
11	4
12	1
13	2
14	0
15	2

Note S5) Relating to Results: Alternative splicing is not enriched on specific chromosomes

Many fungal species have additional small chromosomes that are supernumerary (Akamatsu et al., 1999). They do not contain essential genes and many individuals in the population may not have them, but they exist to adopt additional functions for the organism, like the human Y sex chromosome. Although *S. commune* does not have any supernumerary chromosomes, it could be that the smaller chromosomes are more developed for specific, non-essential or condition specific tasks. It could be argued that genes on these chromosomes, which are highly specialized, are less likely to be alternatively spliced. However, there is no enrichment or depletion for alternatively spliced genes on any chromosome (See Table ST5.1). The number of alternatively spliced genes are not significantly higher or lower on one chromosome than on the others. No chromosome is significantly enriched or depleted for alternatively spliced genes.

Table ST5.1: Odds ratios of enrichment of alternatively spliced genes for each chromosome. Odds ratio and p-values are from a chi-squared test.

Sequence Name	Odds Ratio	p-value
scaffold_1	0.864576	0.029991
scaffold_2	1.11229	0.171928
scaffold_3	1.115113	0.232789
scaffold_4	0.970727	0.713383
scaffold_5	0.989956	0.907507
scaffold_6	0.904298	0.28032
scaffold_7	0.983774	0.847819
scaffold_8	1.043633	0.674506
scaffold_9	0.948184	0.568197
scaffold_10	1.144475	0.24112
scaffold_11	1.021163	0.908819
scaffold_12	1.092326	0.505421
scaffold_13	0.977595	0.836559
scaffold_14	0.810551	0.116395
scaffold_15	1.151552	0.607695
scaffold_16	1.246581	0.337917
scaffold_17	0.873979	0.452997
scaffold_18	1.158336	0.594434
scaffold_20	1.922976	0.558387
scaffold_21	2.991487	0.494098
scaffold_22	0.640172	0.453597
scaffold_23	2.136912	0.701517
scaffold_24	0.427063	0.440541

Note S6) Relating to Figure 2F-G: Alternative splicing events in coding regions affect the reading frame

Alternative splicing may impact the reading frame. For determining whether an event is reading frame neutral, we count the number of events that have a length modulo 3 being zero (see Table ST6.1). Reading frame neutral events are not significantly enriched or depleted in the set of events in coding or non-coding regions. From that we conclude that alternative splicing events have not been driven by evolution to be reading frame neutral.

Table ST6.1: Most alternative splicing events affect the reading frame. CDS/UTR refers to events in coding or UTR regions, respectively, and IR/A5SS/A3SS/ES refers to the four primary events (Figure 1A).

		IR	A5SS	A3SS	ES
CDS	Event RF neutral	523	465	270	18
	Event not RF neutral	1003	839	625	18
UTR	Event divisible by 3	94	75	129	397
	Event not divisible by three	185	163	270	828

Note S7) Relating to Results: Alternative splicing in genes which are read-separable.

The expression of the UTRs of neighboring genes may affect the prediction of alternative transcripts in cufflinks. Little can be done to counter this, but we performed statistics on the small set of 2,516 genes whose neighbors do not extend into the gene boundaries, i.e. read-separable genes where there is zero expression in both the up- and downstream regions of the gene. Prediction of alternative transcripts for these genes is not confounded by neighboring genes. In this set, 207 genes were identified with alternative splicing, similar in frequency and in events found in the total gene set. Exon Skipping (ES) is still the most common event in the UTRs, and Intron Retentions (IR) are the events in coding regions. In tables ST7.1-3, we show the event counts overall, in coding regions and in UTR regions, respectively.

Table ST7.1: Event counts at different levels among read-separable genes.

Event	Event level count	Transcript level count	Gene level count
IR	198	165	145
A5SS	130	124	108
A3SS	87	124	108
ES	157	51	39
MUT	135		18
MA5SS	10		9
MA3SS	7		6

Table ST7.2: Event counts at different levels in the CDS of read-separable genes.

Event	Event level count	Transcript level count	Gene level count
IR	171	144	127
A5SS	108	103	94
A3SS	62	62	56
ES	0	0	0
MUT	0		0
MA5SS	7		7
MA3SS	3		3

Table ST7.3: Event counts at different levels in the UTR of read-separable genes.

Event	Event level count	Transcript level count	Gene level count
IR	27	24	23
A5SS	22	22	20
A3SS	25	25	25
ES	157	51	39
MUT	87		8
MA5SS	1		1
MA3SS	0		0

Note S8) Relating to Results: UTR Terminology

In the example of Figure SF8.1, a gene has two transcripts. Transcripts are composed of a 5’ UTR (UnTranslated Region), a translated region, and a 3’ UTR. In most fungi the length of the UTRs is generally unknown, but splicing events may occur in the UTRs. In this example, there is one region which is spliced out in both transcripts. There can also be alternatively spliced regions inside these unknown UTRs.

There is a specific case in which we can examine alternative splicing in UTRs. When there is an alternative splicing event which disrupts the translated region, RRPM is able to reconstruct splicing events that occur in ‘known’ UTRs that are created by that splicing event. In the example, the second transcript has an intron retention, which induces a frame shift that results in a stop codon. The sequence following this codon (part D of Figure SF8.1) will no longer be translated and is now a known UTR region.

There are therefore two different UTR regions; The unknown and the known UTRs. RRPM can quantify the alternative splicing events that occur only within the known UTRs. A splice junction analysis can give a semi-quantitative analysis of the alternative splicing events that occur in the unknown UTRs.

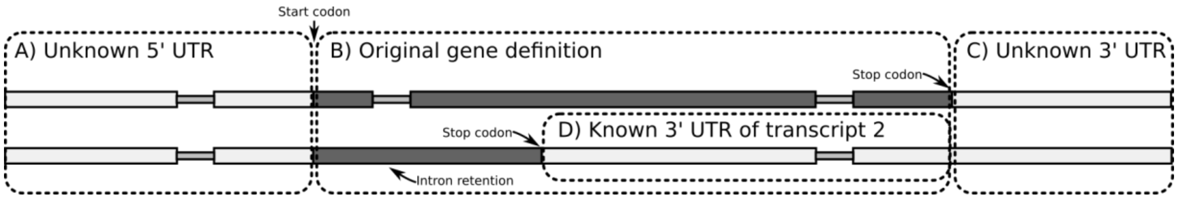

Figure SF8.1: A gene with two transcripts with the up- and downstream UTRs of the original gene definition (A, C), the original gene definition region (B), and the 3’ UTR of the second transcript, whose coding region is shortened due to a premature stop codon introduced by an intron retention (D).

Note S9) Relating to Results: Overview of Splice Junction Analysis

Splice Junction Analysis provides a semi-quantitative overview of alternative splicing across the whole genome. It does not permit an insight into the functional impact of alternative splicing. However, it does allow us to quantify the amount of alternative splicing that RRPM is unable to capture (See supplementary Note S8). Figure S9.1 gives an overview of splice junction analysis.

We classify alternative splicing events in non-coding regions using a process similar to the splice junction analysis of Xie *et. Al*. We describe here our process, which is slightly modified from their work.. Splice junctions indicate the start and end positions of regions on the genome that are spliced out. We aligned RNA-seq of all samples reads to the complete *S. commune* genome using STAR, which produced a splice junction table. From this table, we selected all splice junctions which lay outside annotated gene regions in the reference genome. These splice junctions are clustered based on their overlap. If a cluster contains a splice junction which starts at the same location as a second splice junction, but ends at the same location as a third, then this is an exon skipping event. Based on the number of splice junctions within an exon skipping junction, we counted how many exons were skipped. Following this, as these splice junctions could also be mistaken for alternative 3' and 5' splicing sites, they were removed from the set of splice junctions. The remaining junctions were re-clustered on overlap to search for alternative splicing site events. If a cluster contains multiple splice junctions with the same 5' site, but different 3' sites, then we call a A5SS. Likewise, if a cluster contains multiple splice junctions with the same 3' site, but different 5' sites, then we call a A3SS. As our data is not strand-specific, we cannot with certainty indicate the orientation of reads and we reported only the sum of the number of alternative splicing sites.

The approach used by (Xie et al., 2015) cannot accurately predict intron retentions (IR) due to the density of the genome. A non-zero read coverage in a splice junction is not indicative of an IR, as the coverage may originate from pre-mRNA or the UTR from a neighboring gene. Even if we ignore pre-mRNAs, we will never be able to know if a read supporting a retained intron originates from a gene up stream, or downstream. Therefore, we do not report IR events. Likewise, we are unable to link these alternative events to a single transcript or a gene.

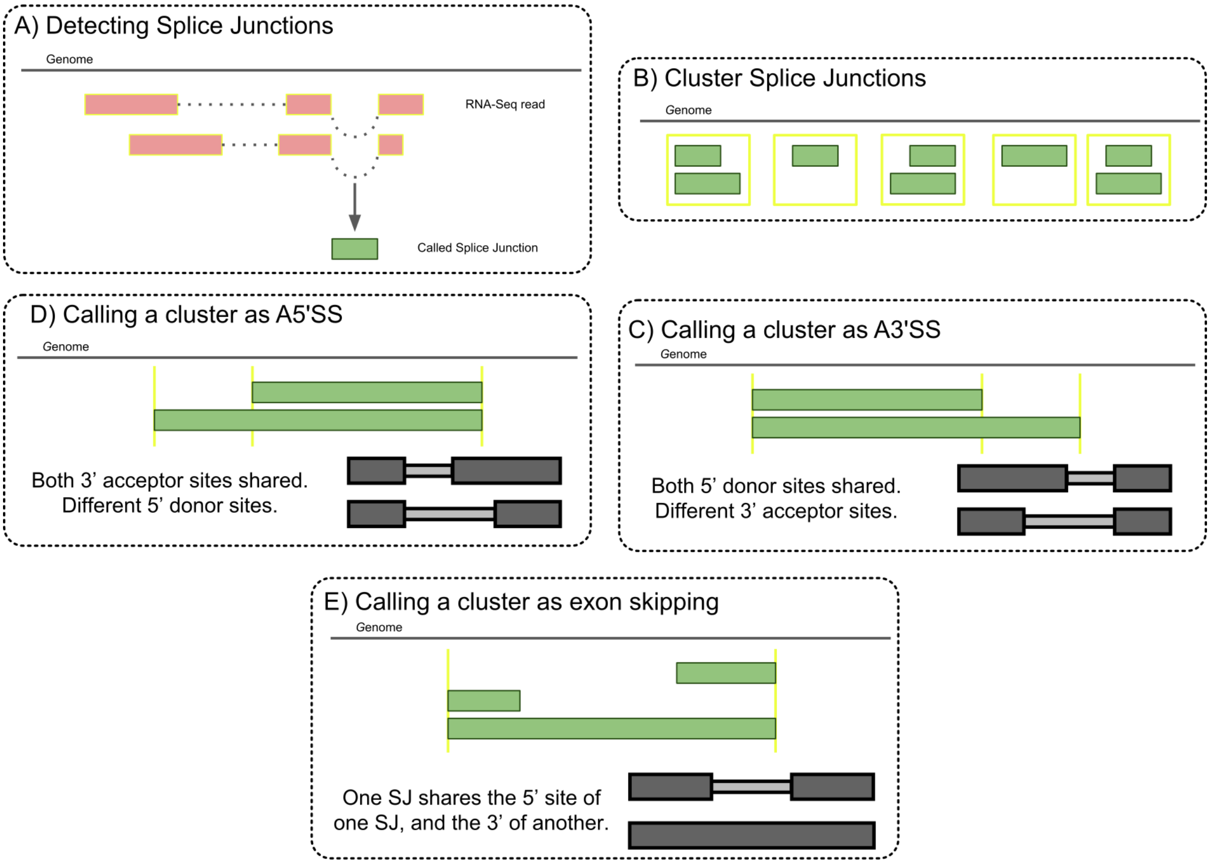

Figure SF9.1: An overview of the splice junction analysis performed in this work. A) We detect splice junctions based on RNA-Seq read alignments to the genome. We select only splice junctions which occur at least twice. B) The splice junctions are then clustered based on their genomic location. If they overlap, they form a cluster. C-E) We call clusters as ES, A5'SS or A3'SS based on the characteristics of the splice junctions in these clusters.

Note S10) Relating to Figure 2F-G: Alternative splicing event counts

In tables ST10.1-3 we give the counts for all events, CDS events and UTR events, respectively.

Table ST10.1: Event counts at different levels.

Event	Event level count	Transcript level count	Gene level count
IR	2033	1726	1569
A5SS	1542	1432	1281
A3SS	1294	1245	1154
ES	1261	559	449
MUT	1381		171
MA5SS	64		63
MA3SS	46		44

Table ST10.2: Event counts at different levels in CDS.

Event	Event level count	Transcript level count	Gene level count
IR	1740	1496	1374
A5SS	1304	1220	1124
A3SS	895	865	821
ES	36	35	33
MUT	12		7
MA5SS	52		52
MA3SS	28		27

Table ST10.3: Event counts at different levels in UTR.

Event	Event level count	Transcript level count	Gene level count
IR	293	270	253
A5SS	238	232	218
A3SS	399	393	376
ES	1225	524	434
MUT	573		65
MA5SS	2		2
MA3SS	1		1

Note S11) Relating to Figure 3B-C: Alternative domain usage

Figures 3B-C in the main text was truncated at 8 annotations, to ease readability. Some genes have more than 8 annotations, and these are provided in figure S11.1.

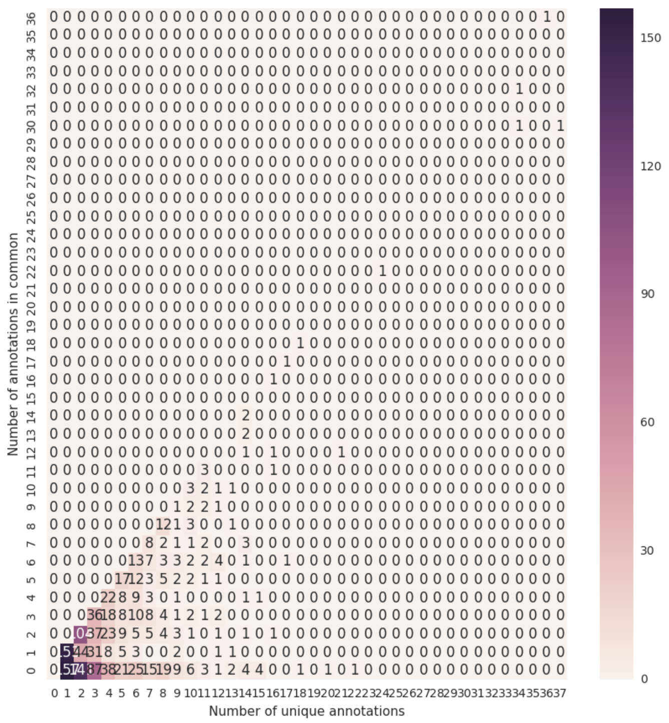

(A)

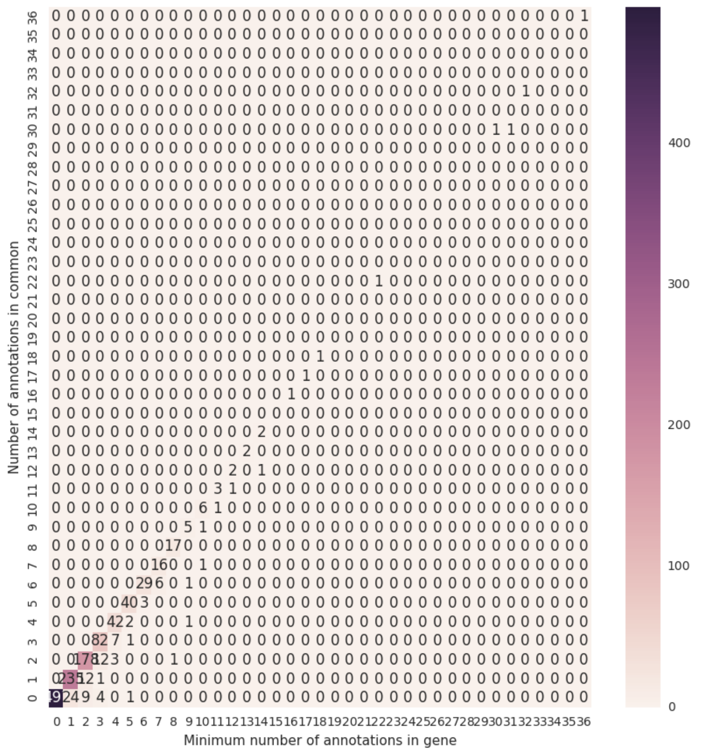

(B)

Figure SF11.1: Implications of alternative splicing on domain predictions. (A) The number of unique annotations are given on the x-axis, and the number of annotations in common between all transcripts is given on the y-axis. If the numbers are not equal, that means that not all transcripts in a gene are annotated with the same domains. In the corresponding cells are given the number of genes with this combination of annotations. Most genes do not exhibit alternative functionality (see diagonal), but quite a number do (see below the diagonal). (B) The smallest number of annotations of a transcript in a gene is given on the x-axis and the number of annotations in common between all transcripts is given on the y-axis. On the diagonal are genes in which the number of annotations in common is limited by a transcript for which annotations are lost due to alternative splicing. Off the diagonal are genes that have enhanced their functional abilities through alternative splicing.

Note S12) Relating to Figure 3D: Alternative subcellular localization

We investigate the effect of alternative splicing on the subcellular location of resultant proteins. In most cases, we are unable to predict alternative subcellular locations (unknown), and in many cases the subcellular location is not affected, but in 71 cases, alternative transcripts' proteins are predicted to exist in different subcellular locations. Figure SF12.1 shows these results.

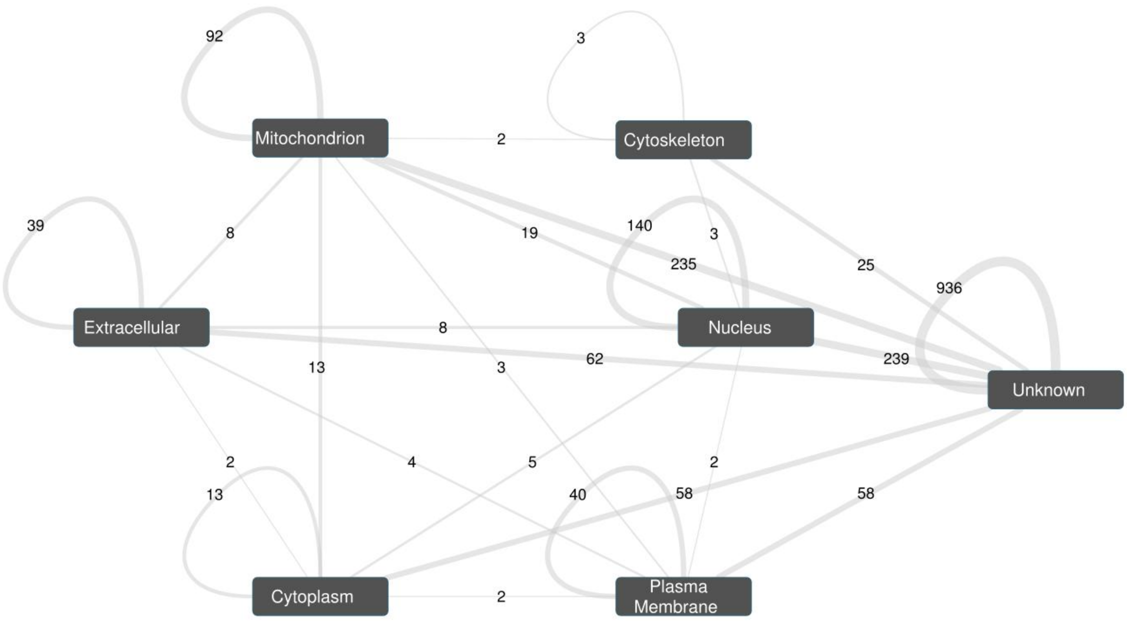

Figure SF12.1: Alternative splicing and subcellular location. Each count on an edge represents a gene which has two alternative splice variants, each of which has a different subcellular location. For example, there are 8 genes wherein one transcript is secreted (extracellular) and the other is transported to the mitochondrion. Self loops indicate that there is no change in a gene's subcellular location. E.g. There are 13 genes where all the transcripts are localized in the cytoplasm. Often, we cannot predict the localization of a transcript, and then the location is called "Unknown".

Note S13) Prevalence of alternative splicing in different functional groups.

We observe alternative splicing in many functional groups that are of interest to *S. commune*. Table ST13.1 indicates the number of genes in each group, and the number of alternatively spliced genes in each group.

Table ST13.1: The number of genes and alternatively spliced genes in selected functional groups.

Functional group	# of genes in group	Overlap with alternatively spliced genes
Transcription Factors	549	93
Cazymes	2058	296
Secreted proteins	775	88
Cytochrome P450s	67	53
Metabolic proteins	642	129

Note S14) Relating to Figure 3A and Results: Alternative transcript activity

Figure 4 in the main text gives an indication of the involvement of alternative splicing in mushroom development, by counting the number of Time Course Switch genes in each time point, where an alternative transcript is more active than the primary transcript. We additionally investigate the activity of these alternatively spliced genes throughout development. Figure SF14.1a shows that the aggregates stage of development always shows a peak of alternative transcript activity, regardless of the expression of these genes; highly and lowly expressed, alternatively spliced genes are differently.

All different functional groups peak in the aggregates stage. Figure SF14.1b shows the number of alternatively spliced genes in each functional category we investigated that peak in each developmental stage. Again, the aggregates stage represents a functional switch for all categories.

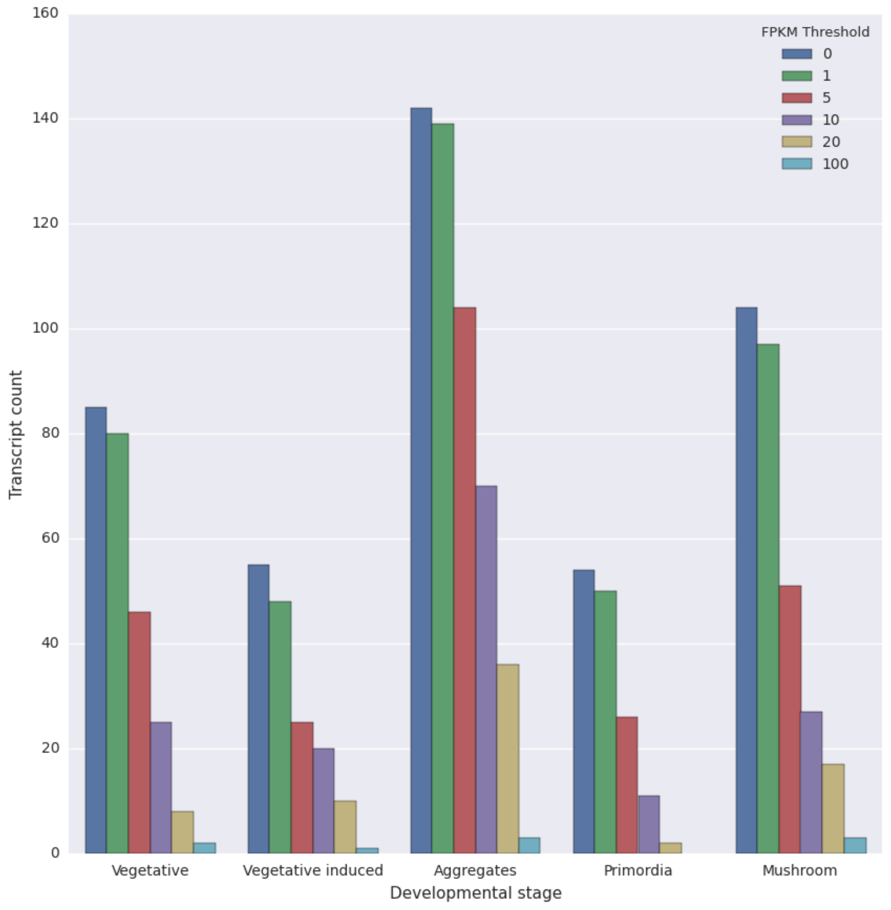

(a)

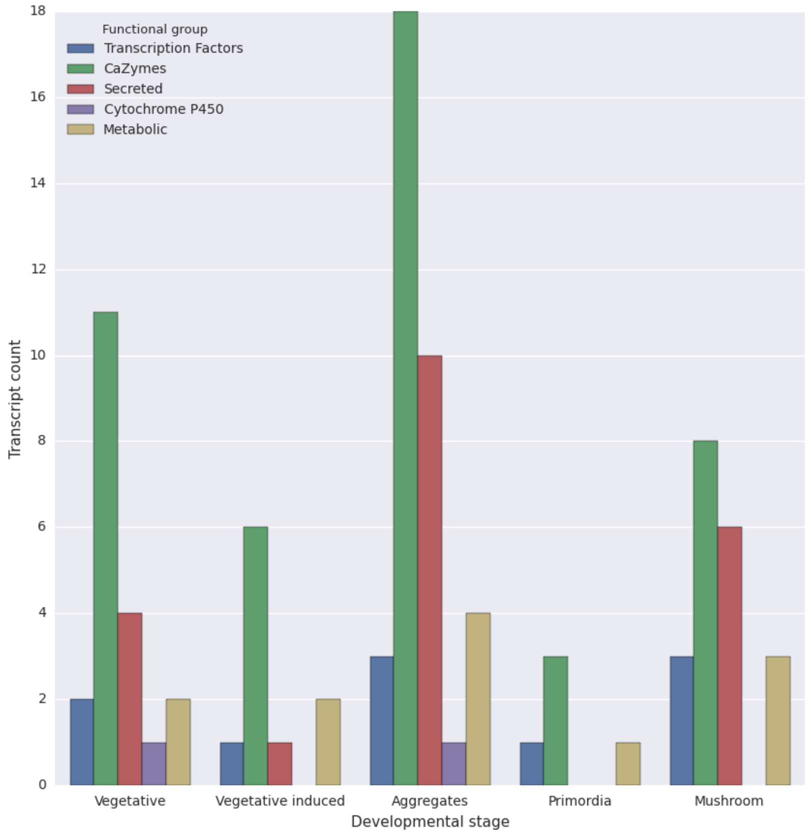

(b)

Figure SF14.1: Alternative transcript activity in developmental stages. (a) Across expression thresholds. (b) Across functional groups.

Note S15) Relating to Results: Named genes in S. commune

As there was no collected list of named genes available, this list was constructed by searching the JGI fungal database for user annotations with the tag 'Name' in both version 2.0 and 3.0. A reciprocal best hit blast search was used to make a mapping from the version 2.0 IDs to the version 3.0 IDs. For the genes for which there was no reciprocal best blast hit, a mapping was done manually and the match with the highest identity was used

Table ST16.1: Named genes in S. commune and their IDs in v3.0. Alternatively spliced genes are given in bold.

Name	JDI ID v3.0
**A-ALPHA Z4**	**2696654**
**AAY4**	**2596714**
ABQ6	2489147
ABR6	2610278
ABS7	2488787
ABT6	2684088
**ABU6**	**2610293**
ABV6	2565082
ADE5	2610442
ALG3	2524870
BAP3-1	2514721
BAP3-3	2514787
BAR3	1034973
**BBP2-1**	**83222**
BBP2-2	2066138
BBP2-5	2066277
BBP2-6	60621
BBP2-7	2514483
BBP2-8	2704857
BBR2	2704852
BETA-FG	2612100
BRI1	2609901
C2H2	1194000
CFS1	12877
CFS2	12877
**CFS3**	**2616150**
CLP1	2580987
CLP2	2613174
CLP3	2570100
CLP4	2635875
**CREA**	**2616742**
DHC1	2609193
DHC2	2743920
DOPA	2174616
DRMIP	2631442
EGC1	2637065

EXP1	2623333
FLP1	2622012
FRT1	2530244
FST3	2629275
FST4	2616096
GAP1	2668143
GAT1	2622228
**HOM1**	**2632356**
HOM2	2634429
**HYD1**	**2703935**
HYD2	2629603
HYD3	2485769
HYD4	2604198
HYD5	2369054
**HYD6**	**2628225**
HYD7	2629420
HYD8	2526471
HYD9	2547619
ICH1	2672639
LCC2	1194451
MFBC	2623183
MIP	2684301
**NIK1**	**2628525**
PAB1	2529077
PKAC1	2484274
PKAC2	2630814
PRI3	2664290
RAS1	2612648
RPB6	2610358
**SC-PA**	**2622148**
SC1	2610513
SC14	2627934
SC15	2625127
SC3	2629632
SC4	2610494
SC6	13059
**SC7**	**2627923**
SCHCO	2613213
SCLCC1	2509814
SCLCC2	1194451
SCMCO1	2621035
SCMCO2	2634619
SCMCO3	2516955
SCMCO4	2483752

**UBI**	**2632518**
UBI4	2744056
WC1	2636923
WC2	2608172

Note S16) Relating to Discussion: Alternative functionality is also present in genes with more than three transcripts

The dikaryotic state represents the fertile state of *S. commune*. Compartments within hyphae contain one nucleus each of both mating partners. This would imply that part of the alternative transcripts may result from unique splicing variants derived from each of the parental nuclei. But, in this study we used isogenic mating partners excluding this possibility. Furthermore, alternative splicing exists also in genes with more than two splice variants.

The two nuclei of *S. commune* are isogenic, and therefore pose no difficulties in the detection of alternatively spliced variants. Nevertheless, we present here results specifically for the set of 227 genes with more than three transcripts, as these alternative transcripts could never be explained by a second nucleus. We find the same trends in the event counts as for the whole set of genes with alternative splicing. Table ST15.1 shows all alternative splicing events in all genes with more than three transcripts, while table ST15.2 and ST15.3 show counts in coding regions and UTR regions, respectively. In this set, 34 genes have alternative subcellular locations. We find 169 genes with time course switches. These genes also have transcript activities peaking at the aggregates stage of development. 7 genes are transcription factors, 26 are predicted cazymes, 6 are predicted secreted proteins, 3 are cytochrome P450s, and 15 are metabolic genes. 5 genes have alternative subcellular locations.

Table ST15.1: Event counts in genes with more than three transcripts.

Event	Event level count	Transcript level count	Gene level count
IR	477	338	200
A5SS	278	247	167
A3SS	186	171	127
ES	265	99	58
MUT	217		21
MA5SS	26		25
MA3SS	22		20

Table ST15.2: Event counts in the coding region of genes with more than three transcripts

Event	Event level count	Transcript level count	Gene level count
IR	390	287	176
A5SS	204	182	133
A3SS	124	116	100
ES	14	14	12
MUT	14		5
MA5SS	17		17
MA3SS	12		11

Table ST15.3: Event counts in UTR regions of genes with more than three transcripts

Event	Event level count	Transcript level count	Gene level count
IR	87	73	57
A5SS	74	73	60
A3SS	62	60	43
ES	251	85	50
MUT	178		19
MA5SS	2		2
MA3SS	1		1

Note S17) Relating to discussion: Kozak consensus sequences in alternatively predicted transcripts.

Using the original gene definitions for *S. commune* v3.0, we constructed a consensus sequence based on ten upstream and ten downstream bases around the start codons (see Table ST17.1). We include a pseudocount in the calculation of the PSSM. We then score each transcript with this PSSM (see Figure SF17.1. We score four different groups: i) the original gene definitions; ii) the alternatively spliced transcript definitions which are the simultaneously the earliest and the longest ORFs; iii) the alternatively spliced transcript definitions which are the longest, but not the earliest; and iv) a set of randomly generated regions around a start codon. We find that all these sets are significantly different from one another (see Table ST17.2). We should note that because the PSSM was learned with the original gene definitions, that this will of course give other transcription start sites a worse score.

Table ST17.1: The kozak consensus PSSM for *S. commune*. The scores are the –log fraction of each base at that location.

	A	C	G	T	
-10	1.549535	1.085726	1.512794	1.470968	C
-9	1.558256	1.064673	1.567358	1.443666	C
-8	1.675058	0.844779	1.815499	1.512794	C
-7	1.579316	1.075983	1.401219	1.576851	C
-6	1.429227	1.170156	1.464622	1.518582	C
-5	1.788522	0.831696	1.82413	1.443397	C
-4	1.689073	0.734224	1.669641	1.916529	C
-3	0.791195	1.886307	1.245889	2.23137	A
-2	1.570105	0.786717	1.991435	1.608739	C
-1	1.841217	0.711562	1.467928	2.119459	C
A	0.000191	9.663261	9.663261	9.663261	A
T	9.663261	9.663261	9.663261	0.000191	T
G	9.663261	9.663261	0.000191	9.663261	G
+1	1.648926	1.415779	1.136712	1.410034	G
+2	1.610647	0.73967	1.910926	1.742815	C
+3	2.12264	0.915909	1.334327	1.528794	C
+4	1.47485	1.390435	1.159762	1.567053	G
+5	1.502172	0.962914	1.702938	1.544458	C
+6	1.964779	0.789793	1.447714	1.767453	C
+7	1.395043	1.259461	1.262377	1.685293	C/G
+8	1.429493	1.022082	1.777556	1.462424	C
+9	1.754874	0.949679	1.393504	1.650249	C
+10	1.404839	1.221439	1.267784	1.724103	C/G

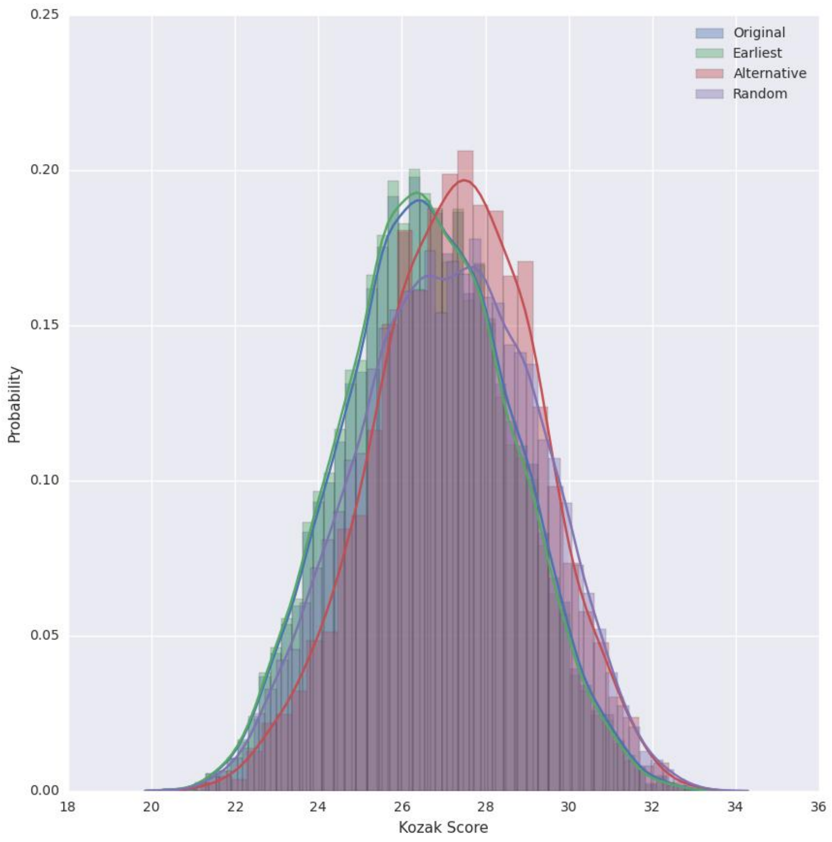

Figure SF17.1: Distribution of PSSM scores (using PSSM in table 19) for different sets of kozak sequences. We score the original set of gene definitions (blue). For alternatively spliced genes, we score the transcripts which have the earliest ORFs (green), and the transcripts which are the largest, but not the earliest (red) separately. We also score a set of randomly generated sequences around a start codon (purple). Because we use here a sum of –log values, lower scores are better.

Table ST17.2: Differences in Kozak distributions between the different sets of transcripts: above diagonal) p-values for a two-sample t-test; below the diagonal) p-values for a two-sample Kolmogorov-Smirnov test. All tests are significantly different (α=0.05).

	Original	Earliest	Alternative	Random
Original	-	3.32712317e-003	1.51166876e-066	1.49239588e-115
Earliest	7.93097214e-002	-	1.71706779e-079	1.54486194e-144
Alternative	2.86769421e-49	2.19535049e-58	-	1.65060923e-06
Random	2.15830126e-090	2.22506026e-112	3.22309472e-010	-

Note S18) Relating to Experimental Procedure: The S. commune experimental design

RNA of colonies of wildtype H4-8 was sampled at different time points (Figure SF18.1). The dikaryotic knockout strains of eight transcription factor genes and one signaling protein were sequenced too. Some knockouts are arrested at a particular stage of development. They were sampled at the time when a wildtype mushroom reached the aggregate stage, and again when the same wildtype reached the mushroom stage. Those knockouts which do (at some point) form aggregates and or mushrooms were sampled at those stages, regardless how long it took them to reach these stages (see figure SF18.1)

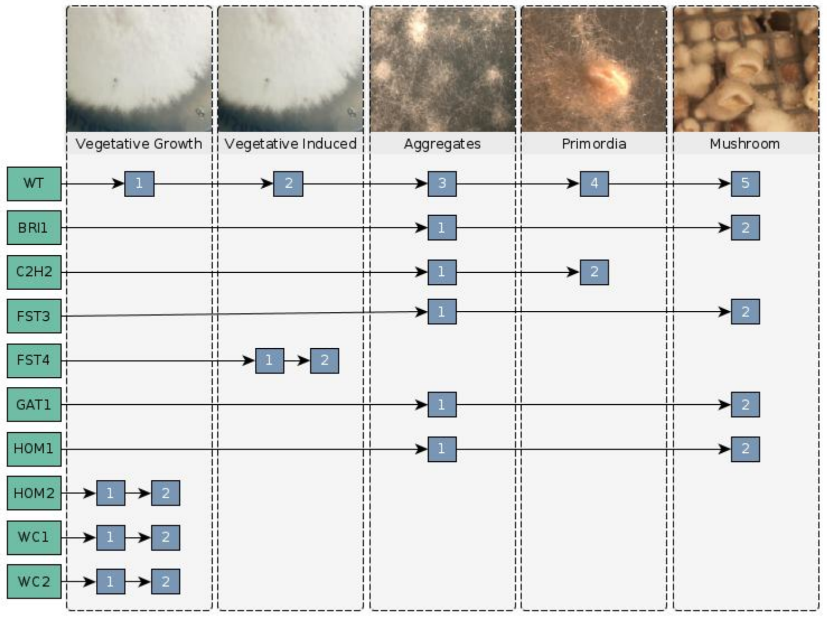

Figure SF18.1: The *S. commune* RNA-Seq dataset. In addition to the wildtype samples at the developmental stages, we also isolated mRNA from nine dikaryotic deletion strains [Ohm2010b,Ohm2013]Δwc-1Δwc-1, Δwc-2Δwc-2, Δhom1Δhom1, Δhom2Δhom2, Δfst3Δfst3 Δfst4Δfst4, Δbri1Δbri1, Δgat1Δgat1, and Δc2h2Δc2h2 at two time points. The first was sampled at the same time as a simultaneously grown wildtype sample reaches the aggregates stage of development, and the second when the wildtype reaches a mature mushroom.

Note S19) Relating to Experimental Procedure: Region restricted Probabilistic Modeling

Region Restricted Probabalistic Modeling (Figure SF19.1) was designed to prevent the influence of overlapping UTRs on isoform reconstruction. RNA-Seq reads are aligned to a transformed genome that has the intergenic regions removed. Based on the reads that align within each gene region, cufflinks is run with Reference annotation based transcript (RABT) mode to predict transcripts. The predicted transcripts are filtered. For each gene, the remaining transcripts are used to construct consensus structures, which are used to call alternative splicing events.

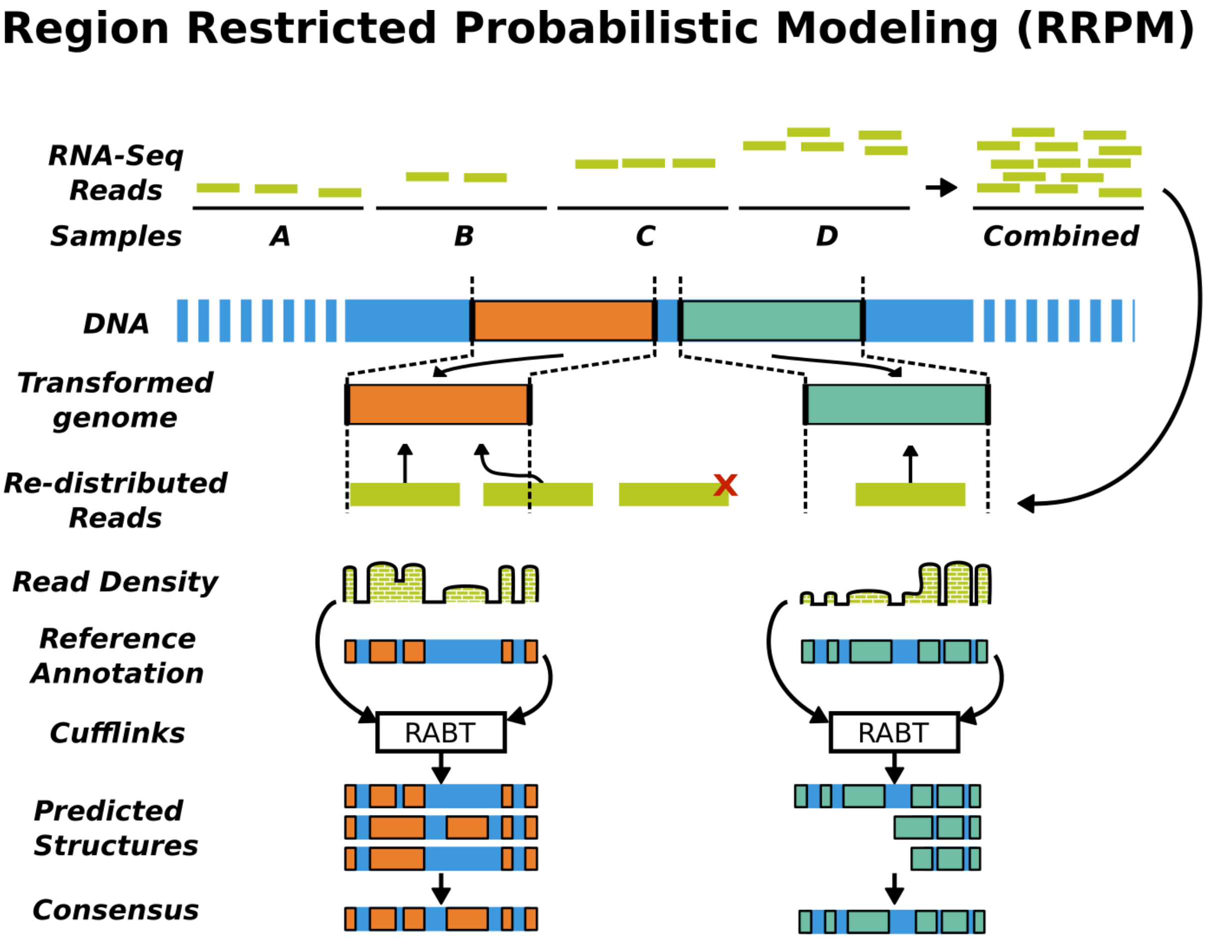

Figure SF19.1: A schematic overview of the RRPM method. Reads from all RNA-Seq samples are combined into one super-sample. These reads are then aligned to a transformed genome, where regions between known gene coding regions are removed. Reads which do not align within these gene coding regions are thereby discarded. The aligned reads are given to Cufflinks, together with the annotated genes, to predict structures. These predicted alternative structures are filtered to produce a set of transcripts that are used to construct a consensus structure.

Note S20) Relating to Experimental Procedure: Tool and algorithm parameters

Parameters used for each tool in the analysis.

Table ST20.1: Parameters used for tools

Tool	Option	Value
TRIMMOMATIC 0.32	LEADING	3
	TRAILING	3
	SLIDINGWINDOW	4:15
	MINLEN	36
STAR 2.4.0f1	--alignIntronMax	5000
	--alignIntronMin	10
CUFFLINKS 2.1.1 (with BOOST 1.53.0 and eigen 3.1.2)	--max-intron-length	5000
	--min-intron-length	25
	--overlap-radius	25
	--max-bundle-length	250000
CUFFDIFF 2.1.1	--upper-quartile-norm	
	--max-bundle-frags	100000000000
INTERPROSCAN 5.13-52.0	-iprlookup	
	-goterms	
	-b	
Python 2.7.10		
Ipython 2.1.0		
Ibidas 0.1.26		
Numpy 1.9.2		
Scipy 0.16.0 [Jones2001]		
Bx_python 0.7.3		
TargetP 1.1	-N	
SignalP 4.1	-t	euk
TMHMM 2.0c		
Wolf PSort 0.2		

Note S21) Relating to Experimental Procedure: Effects of each filtering step on the number of predicted transcripts

The filtering pipeline removes structures that do not meet our strict criteria. The original annotation contained 16,319 genes with one transcript each, Cufflinks (Trapnell et al., 2010) produces predicted transcripts. These predicted transcripts are filtered based on various criteria. Table ST21.1 indicates the number of transcripts and genes that are removed as a result of each filtering step.

Table ST21.1: The number of genes and transcripts after each filtering step in *S. commune*. Full read support refers to the requirement that all splice junctions in a transcript are supported by RNA-Seq data, and coverage filtering means that there must be a non-zero expression of the transcript.

Step in Pipeline	Number of Genes	Number of Transcripts
Original annotation	16,319	16,319
Cufflinks output	16,319	25,304
Strand filtering	16,319	23,951
Full Read Support / Coverage filtering	13,136	15,836
ORF filtering	12,988	15,522

Note S22) Relating to Experimental Procedure: Construction of consensus-based reference annotation per gene

Instead of using the reference annotation as reference we create a reference annotation based on all observed transcripts for a gene, without taking into consideration the reference. Schematically this is shown in Figure SF22.1.

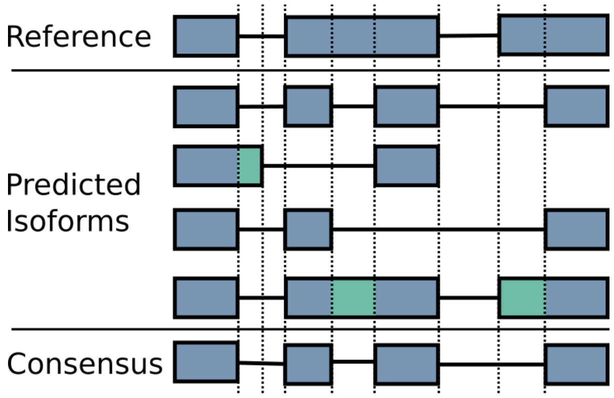

Figure SF22.1: Consensus gene annotation based on expressed transcripts. Boxes indicate exons. Filled lines indicate connections between exons. Dashed vertical lines indicate exon boundaries in the consensus. Blue regions indicate regions that conform to the consensus, and turquoise regions indicate exon regions that differ from the consensus. In this example, the original reference annotation described a second exon, which in actuality is two exons with an intron retention. Comparing the predicted structures to this reference would result in the peculiar, unclear situation of having to define this case. By comparing our predicted transcripts to the consensus structure, we resolve this issue.

The consensus is constructed using an Interval Tree, which organizes intervals, or regions in such a way that one can easily search the tree for intervals which overlap with another interval. The consensus is described in **Error! Reference source not found.**SA22.1.

Algorithm SA22.1: The construction of a consensus structure

```
1. Collect all exon regions for all isoforms in one list, including
   duplicates.
2. Sort the exon regions by size
3. Create an interval tree
4. For each exon region, from smallest to largest:
      a. Check overlap of this exon in the region tree
      b. If the exon region does not overlap
                  • Add exon region to the exon tree
      c. If the exon region overlaps with one exon
                  • This is an exon with a 3 or 5' alternative SS. Extend
                    the 3' and 5' ends of the exon in the tree by the
                    largest boundary of the two exons. Maintain a list of
                    all alternative boundaries.
      d. If the exon region overlaps with more than one exon
                  • This is an intron retention. Extend the 5' end of the
                    leftmost exon in the retention, and the 3' end of the
                    rightmost exon by the longest boundary for the exons,
                    respectively. Maintain a list of all alternative
                    boundaries.
5. For each region in the interval tree, choose the most occurring
   boundaries. If there is no most occurring region, choose the largest
   one.
6. Return the regions in the interval tree
```

Note S23) Relating to Experimental Procedure: Calling alternative splicing events

We call events on an individual transcript by the algorithm described in Algorithm SA23.1.

Algorithm SA23.1: Calling the events in a transcript relative to a consensus.

```
1. Construct an interval tree using the exon regions in the consensus
   structure.
2. Set variable exons_found = 0
3. For each exon in the isoform
      a. Check the overlap to the consensus tree
      b. Increase variable exons_found by overlap
      c. If the overlap is one
            • If the start of the exon is not the same
                  • Mark exon as A5'SS
            • If the end of the exon is not the same
                  • Mark exon as A3'SS
      d. If the start and end of the exon are the same
            • Mark exon as identical to consensus
      e. If the overlap is greater than one
            • Mark as intron retention
4. For each exon which was NOT found in the interval tree
      • Mark exon as skipped
```

Note S24) Relating to Experimental Procedure: Prediction of Transcription factors

Table ST24.1 lists the interpro domains that were used to predict transcription factors.

Table ST24.1: Domain annotations used to predict transcription factors.

Domain Type	Domain ID	Description
bHLH	IPR001092	Basic helix-loop-helix dimerisation region bHLH
bZIP	IPR004827	Basic-leucine zipper (bZIP) transcription factor
bZIP	IPR011616	bZIP transcription factor, bZIP_1
bZIP	IPR011700	Basic leucine zipper
C2H2 zinc finger	IPR007087	Zinc finger, C2H2-type
Forkhead	IPR001766	Fork head transcription factor
GATA type zinc finger	IPR000679	Zinc finger, GATA-type
Homeobox	IPR000747	'Homeobox' engrailed-type protein
Homeobox	IPR001356	Homeobox
Homeobox	IPR001827	Homeobox protein, antennapedia type
Homeobox	IPR003120	Transcription factor, STE-like
Homeobox	IPR003350	Homeodomain protein CUT
Homeobox	IPR003654	Paired-like homeodomain protein, OAR
Homeobox	IPR003893	Iroquois-class homeodomain protein
Homeobox	IPR006455	Homeobox domain, ZF-HD class
Homeobox	IPR007103	POU homeobox
Homeobox	IPR007104	Paired-like homeobox
Homeobox	IPR007106	SIX/SINE homeobox
Homeobox	IPR007107	LIM homeobox
Homeobox	IPR007738	Homeoboxprospero-like
MADS-box	IPR002100	Transcription factor, MADS-box
Myb	IPR001005	Myb, DNA-binding
Zn2Cys6	IPR001138	Fungal transcriptional regulatory protein, N-terminal
Zn2Cys6	IPR007219	Fungal specific transcription factor
APSES	IPR003163	DNA-binding, yeast
HMG	IPR000079	High mobility group protein HMG14 and HMG17
HMG	IPR000116	High mobility group proteins HMG-I and HMG-Y
HMG	IPR000135	High mobility group proteins HMG1 and HMG2
HMG	IPR000637	HMG-I and HMG-Y, DNA-binding
HMG	IPR000910	HMG1/2 (high mobility group) box
HMG	IPR009071	High mobility group box
Heteromeric CCAAT factors	IPR001289	CCAAT-binding transcription factor, subunit B
Heteromeric CCAAT factors	IPR003956	Histone-like transcription factor CBF/NF-Y/archaeal histone, subunit A
Heteromeric CCAAT factors	IPR003958	Transcription factor CBF/NF-Y/archaeal histone
Grainyhead/CP2	IPR007604	CP2 transcription factor
TEA/ATTS	IPR000818	TEA/ATTS
Centromere protein B, DNA-binding region	IPR006600	Centromere protein B, DNA-binding region
NDT80/PhoG like DNA-binding	IPR007888	NDT80/PhoG like DNA-binding
Nucleic acid-binding, OB-fold	IPR008994	Nucleic acid-binding, OB-fold
Winged helix repressor DNA-binding	IPR011991	Winged helix repressor DNA-binding
Transcription factor jumonji	IPR013129	Transcription factor jumonji

YL1 nuclear protein	IPR008895	YL1 nuclear
WRKY DNA-binding domain	IPR003657	DNA-binding WRKY
UAF complex subunit Rrn10	IPR007898	UAF complex subunit Rrn10
Negative transcriptional regulator	IPR007396	Negative transcriptional regulator
ssDNA-binding transcriptional regulator	IPR009044	ssDNA-binding transcriptional regulator
Tubby transcription factors	IPR000007	Tubby
Tubby transcription factors	IPR005398	Tubby protein, N-terminal
SGT1	IPR010770	SGT1
SART1	IPR005011	SART-1 protein
RFX DNA-binding domain	IPR003150	DNA-binding RFX
p53-like transcription factor	IPR008967	p53-like transcription factor, DNA-binding
p53-like transcription factor	IPR012346	p53 and RUNT-type transcription factor, DNA-binding
Zinc finger, NF-X1-type	IPR000967	Zinc finger, NF-X1-type
Zinc finger, MIZ-type	IPR004181	Zinc finger, MIZ-type
Mating-type protein MAT alpha 1	IPR006856	Mating-type protein MAT alpha 1
Zinc finger, LSD1-type	IPR005735	Zinc finger, LSD1-type
Cold-shock protein, DNA-binding	IPR002059	Cold-shock protein, DNA-binding
AT-rich interaction region	IPR001606	AT-rich interaction region
Helix-turn-helix, AraC type	IPR000005	Helix-turn-helix, AraC type
Bacterial regulatory protein GntR, HTH	IPR000524	Bacterial regulatory protein GntR, HTH
Bacterial regulatory protein, LacI	IPR000843	Bacterial regulatory protein, LacI
Bacterial regulatory protein, LuxR	IPR000792	Bacterial regulatory protein, LuxR
Zinc finger, BED-type predicted	IPR003656	Zinc finger, BED-type predicted
CCR4-Not complex component, Not1	IPR007196	CCR4-Not complex component, Not1
DDT	IPR004022	DDT
Transcription factor E2F/dimerisation partner (TDP)	IPR003316	Transcription factor E2F/dimerisation partner (TDP)
GCN5-like 1	IPR009395	GCN5-like 1
Zinc finger, GRF-type	IPR010666	Zinc finger, GRF-type
Helix-turn-helix type 3	IPR001387	Helix-turn-helix type 3
Helix-turn-helix, Psq	IPR007889	Helix-turn-helix, Psq
Homeodomain-like	IPR009057	Homeodomain-like
Iron dependent repressor	IPR001367	Iron dependent repressor
Lambda repressor-like, DNA-binding	IPR010982	Lambda repressor-like, DNA-binding
Bacterial regulatory protein, MarR	IPR000835	Bacterial regulatory protein, MarR
Zinc finger, PARP-type	IPR001510	Zinc finger, PARP-type
Zinc finger, DHHC-type	IPR001594	Zinc finger, DHHC-type
Zinc finger, CCHC-type	IPR001878	Zinc finger, CCHC-type
Zinc finger, Rad18-type	IPR006642	Zinc finger, Rad18-type putative

putative		
Bacterial transcription activator, effector binding	IPR010499	Bacterial transcription activator, effector binding
Transcription factor TFIIS	IPR001222	Transcription factor TFIIS
Pathogenesis-related transcriptional factor and ERF	IPR001471	Pathogenesis-related transcriptional factor and ERF
Bromodomain transcription factor	IPR006565	Bromodomain transcription factor

References

Akamatsu, H., Taga, M., Kodama, M., Johnson, R., Otani, H., and Kohmoto, K. (1999). Molecular karyotypes for Alternaria plant pathogens known to produce host-specific toxins. Curr. Genet. *35*, 647–656.

Letunic, I., and Bork, P. (2011). Interactive Tree Of Life v2: online annotation and display of phylogenetic trees made easy. Nucleic Acids Res. *39*, W475–W478.
